# Murine Oviductosomes (OVS) microRNA profiling during the estrous cycle: Delivery of OVS-borne microRNAs to sperm where *miR-34c-5p* localizes at the centrosome

**DOI:** 10.1038/s41598-018-34409-4

**Published:** 2018-10-31

**Authors:** Zeinab Fereshteh, Skye A. Schmidt, Amal A. Al-Dossary, Monica Accerbi, Cecilia Arighi, Julie Cowart, Jia L. Song, Pamela J. Green, Kyungmin Choi, Soonmoon Yoo, Patricia A. Martin-DeLeon

**Affiliations:** 1 Department of Biological Sciences, University of Delaware, Newark, DE 19716 USA; 2 Department of Plant and Soil Sciences, Delaware Biotechnology Institute, University of Delaware, Newark, DE 19711 USA; 3 Present Address: Department of Biology, College of Medicine, Imam Abdulrahman Bin Faisal University, P.O. Box 1982, Dammam, 31441 Saudi Arabia; 4 Center for Bioinformatics and Computational Biology, University of Delaware, Newark, DE 19711 USA; 5 A.I. DuPont Hospital for Children, 1600 Rockland Rd, Wilmington, Delaware, 19803 USA

## Abstract

Oviductosomes (OVS) are nano-sized extracellular vesicles secreted in the oviductal luminal fluid by oviductal epithelial cells and known to be involved in sperm capacitation and fertility. Although they have been shown to transfer encapsulated proteins to sperm, cargo constituents other than proteins have not been identified. Using next-generation sequencing, we demonstrate that OVS are carriers of microRNAs (miRNAs), with 272 detected throughout the estrous cycle. Of the 50 most abundant, 6 (12%) and 2 (4%) were expressed at significantly higher levels (P < 0.05) at metestrus/diestrus and proestrus/estrus. RT-qPCR showed that selected miRNAs are present in oviductal epithelial cells in significantly (P < 0.05) lower abundance than in OVS, indicating selective miRNA packaging. The majority (64%) of the top 25 OVS miRNAs are present in sperm. These miRNAs’ potential target list is enriched with transcription factors, transcription regulators, and protein kinases and there are several embryonic developmentally-related genes. Importantly, OVS can deliver to sperm miRNAs, including *miR-34c-5p* which is essential for the first cleavage and is solely sperm-derived in the zygote. Z-stack of confocal images of sperm co-incubated with OVS loaded with labeled miRNAs showed the intracellular location of the delivered miRNAs. Interestingly, individual miRNAs were predominantly localized in specific head compartments, with *miR-34c-5p* being highly concentrated at the centrosome where it is known to function. These results, for the first time, demonstrate OVS’ ability to contribute to the sperm’s miRNA repertoire (an important role for solely sperm-derived zygotic miRNAs) and the physiological relevance of an OVS-borne miRNA that is delivered to sperm.

## Introduction

The oviductal luminal fluid is one of the reproductive secretions in which extracellular vesicles (EVs), known to play a role in cell-to-cell communication^1,2^, are present^3,4^. In the mouse where oviductal EVs (or oviductosomes, OVS) were first detected^3^ and shown to arise by the apocrine pathway^5^, they were shown to be of both exosomal (50–100 nm) and microvesicular (100–1000 nm) sizes^3,5^. OVS have now also been reported in bovine oviductal secretions (*in vivo*) and in media of cultured bovine epithelial cells^6–8^, as well as in human oviductal fluid^5^. In these two species and in the mouse, proteins have been identified in OVS cargoes either on the basis of proposed candidate approach from known cargo constituents of epididymosomes and prostasomes, as in the case of Plasma membrane Ca^2+^-ATPase 4 (PMCA4)^9,10^, or from proteomic analysis^7^. Generally, in addition to proteins, a variety of RNAs, including mRNAs and microRNAs (miRNAs) which are major regulatory components, have been shown to be present in the cargoes of EVs^1,11^. Further, these RNAs are known to be transferred to target cells to alter gene expression^1,2^. Because of the importance of miRNAs in the regulation of endogenous gene expression, it is important to determine if OVS carry them in their cargoes and serve as a means of transporting them to target cells, such as sperm. However, to date, miRNA expression in OVS has not been reported for any of the three species in which these EVs have been identified.

It is known in humans^12,13^, bovine^14^ and equine^15^ that a large number of miRNAs are present in the follicular fluid and these impact a variety of pathways including the MAPK, WNT, and TGFβ^16^. In bovine, an impressive cargo of miRNAs have been shown to exist in EVs in the epididymal luminal fluid, the epididymosomes, where individual signatures are found for those derived from the caput and the caudal regions^17^. In murine epididymosomes a variety of non-protein coding small RNAs were shown to be present^18^, as well as a complex profile of miRNAs^19^.

Importantly, it has been shown that several miRNA species in murine epididymosomes could be transferred to sperm, revealing a potential mechanism for the modification of the sperm epigenome^19^. MiRNAs have also been found in the cargoes in EVs released in human uterine flushings and *in vitro* from human cultured endometrial epithelial cells^20^. EVs secreted *in vivo* in the uterine fluid (uterosomes) of sheep encapsulate a large number of both miRNAs and mRNAs^21^. Significant differences were found between the uterosomal miRNA cargoes of pregnant and non-pregnant sheep, and the study supported the hypothesis that uterosomes can deliver endogenous beta retroviruses (enJSRVs) RNA to the ovine conceptus where they regulate trophectoderm development^21^. The present study tested the hypothesis that OVS carry miRNAs during the murine estrous cycle, and that specific miRNAs in the estrus oviductosomal cargo can be delivered to sperm during their communication with the oviduct.

## Results

### OVS are isolated and structurally characterized by TEM and Western blotting

**Figure 1:**
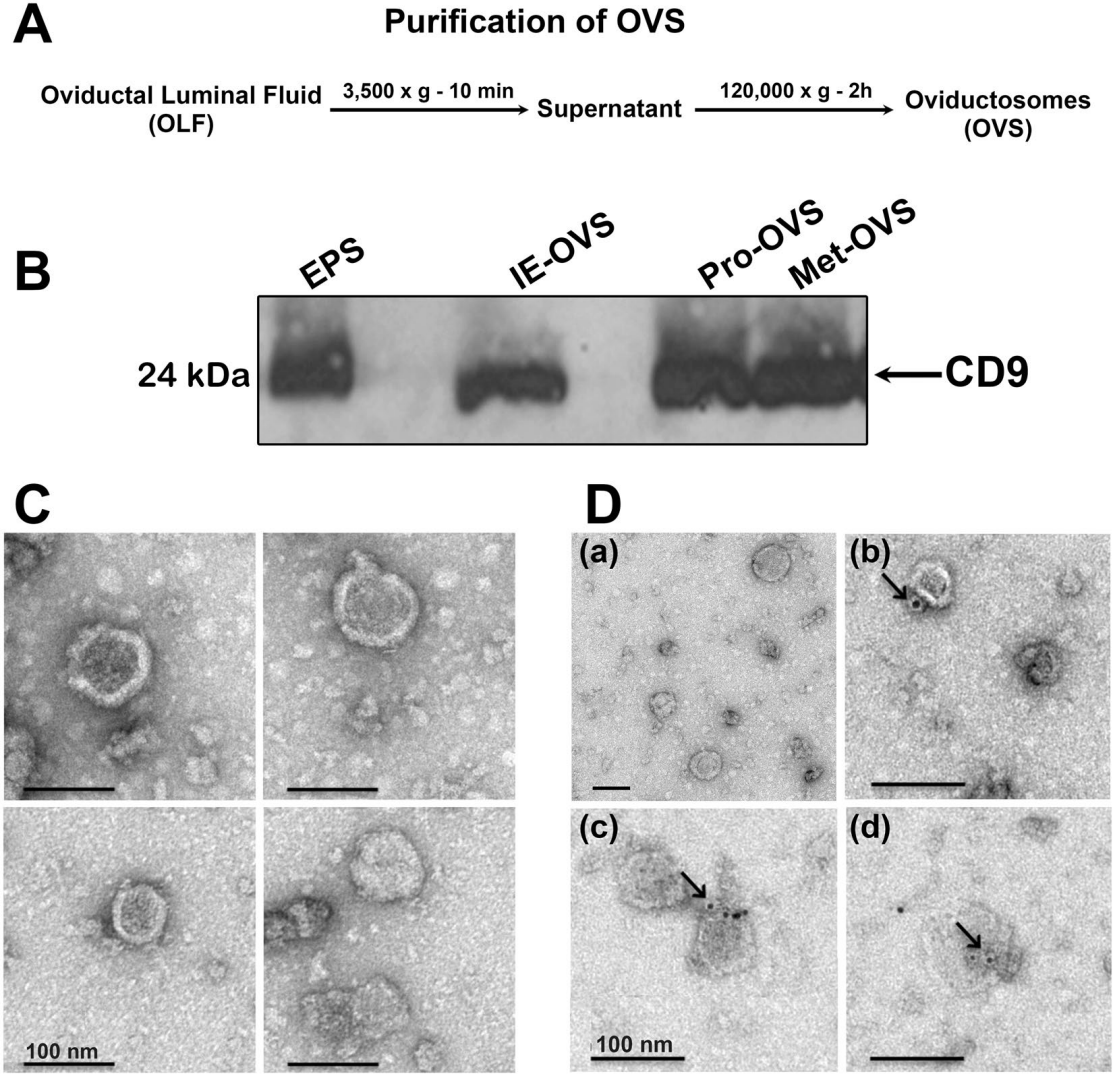
Characterization of OVS isolated from oviductal fluids (OLF). (**A**) Protocol for isolation of oviductosomes (OVS) from OLF using ultracentrifugation. (**B**) Western blot reveals the presence of CD9 (24 kDa) on OVS from proestrus (Pro-OVS), metestrus (Met-OVS), and induced estrus (IE-OVS), as well as epididymosomes (EPS) used as a positive control. Each lane contains 40 μg of protein (n = 3). (**C**) Negative staining and TEM of OVS show the presence of membranous vesicles of both exosomal (<100 nm) and microsomal (100 nm–1 µm) sizes. (**D**) Immunogold labeling (6 nm gold particles) of CD9 is shown in OVS. Gold particles on individual OVS are seen arrowed on the exterior of the membrane (D-b-d). In the absence of primary antibodies and the presence of mouse IgG, gold particles were absent (**D-a**), indicating the specificity of the antibody. Scale bar = 100 nm.

An ultracentrifugation method was applied for isolation and purification of murine OVS (Fig. 1A). This technique can isolate enriched OVS populations with high recovery which can generate appropriate material for comprehensive endpoint analysis of OVS cargo^3,5,22^. Transmission electron microscopy with negative staining (Fig. 1C) and Western analysis (Fig. 1B) revealed that the purified EVs isolated from oviductal luminal fluid (OLF) of various stages of estrous had the characteristic sizes and biochemical marker of OVS. Figure 1B shows the presence in OVS of the 24 kDa CD9 tetraspanin whose extracellular domain is known as a biochemical marker of exosomal membranes^1,23^. The presence of CD9 was confirmed by immunolabeling (Fig. 1D), attesting to the enrichment and purity of OVS in the preparations.

### OVS carry similar repertoires of miRNAs throughout the estrous cycle where the majority have unchanged expression levels at Pro/Est and Met/Diest

**Figure 2:**
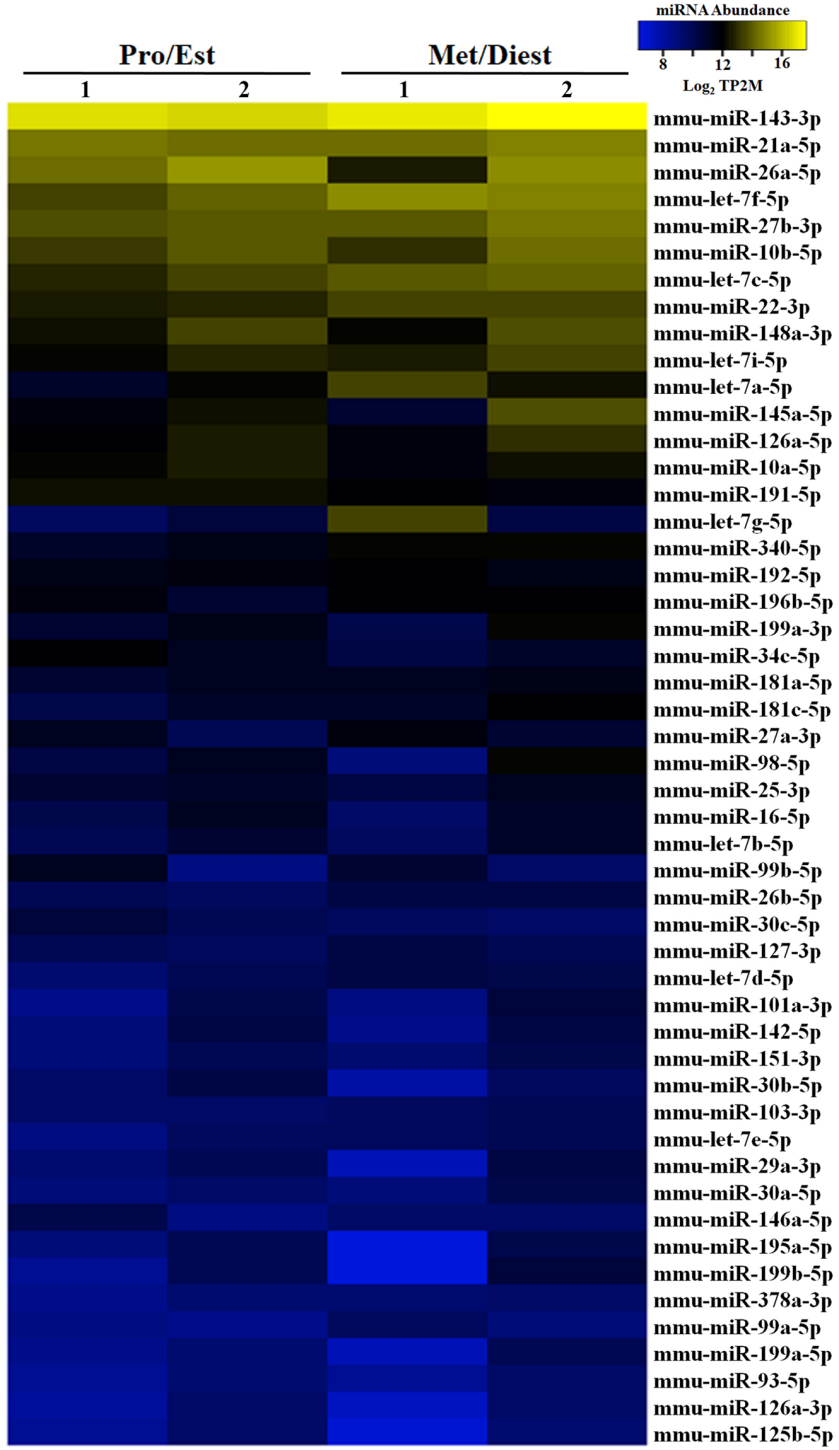
Heat map with hierarchal clustering analysis of the top 50 most abundant miRNAs in purified OVS from females in Pro/Est and Met/Diest. Analysis was performed on data from two biological replicates of each sample. Yellow shading represents miRNA abundance higher than 10 transcripts per two million (10TP2M), and blue shading represents miRNA abundance between 6 and 10 TP2M. MiRNA abundance in Pro/Est and Met/Diest is consistent between samples for 88% of the miRNAs.

**Figure 3:**
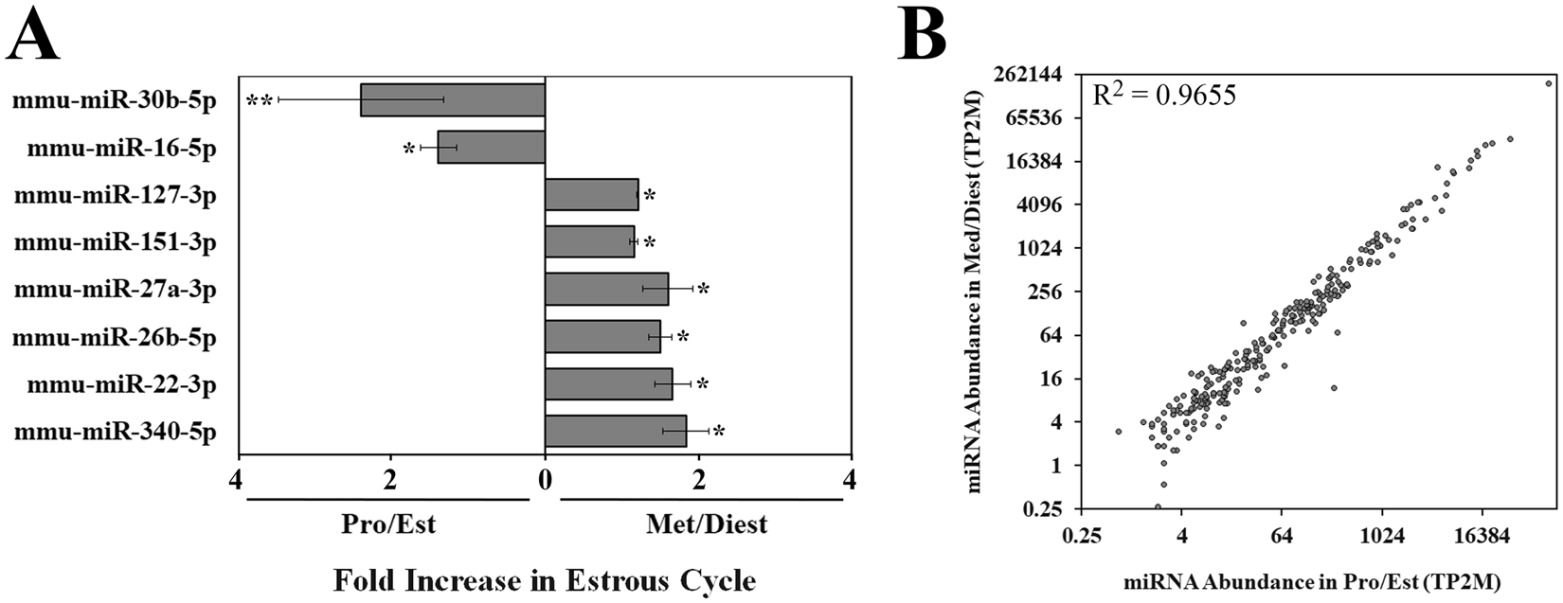
Generally, miRNA expression levels in OVS are similar in Pro/Est and Met/Diest samples. (**A**) Eight miRNAs in the top 50 are present in different abundances in Pro/Est and Met/Diest samples. Six (12%) miRNAs are significantly more abundant in Met/Diest, and 2 (4%) miRNAs are significantly more abundant in Pro/Est, with a fold change ≥ 1.5. (^*^P < 0.05, ^**^P < 0.01). (**B**) MiRNA abundance is correlated between Pro/Est and Met/Diest samples. Data are from 272 miRNAs with abundances of at least 2TP2M in both biological replicates of either Pro/Est or Met/Diest samples. Data shown are from Biorep 2 (R^2^ = 0.9655). Biorep 1 (not presented) shows a similar pattern with R^2^ = 0.9486.

Next-generation sequencing was used to characterize the miRNA cargo present in oviductal EVs (OVS) collected during proestrus/estrus (Pro/Est) and metestrus/diestrus (Met/Diest) stages. The data were collected using >80 females in two biological replicates. The replicates were consistent in sequencing depth, size profile, and miRNA sequence abundance (Supplementary Fig. 1S). This approach identified 272 miRNAs with an abundance of at least 2 transcripts per 2 million (2TP2M) in both biological replicates from pooled Pro/Est or Met/Diest OVS (Supplementary Table S1). The top 50 miRNAs with the highest abundance are seen in Fig. 2. Of these, 8 or 16% showed differential expression with significantly higher levels seen for 6 miRNAs (12%) in Met/Diest (*P < 0.05) and 2 miRNAs (4%) in Pro/Est. (**P < 0.01; *P < 0.05), with a fold change ≥ 1.5 (Fig. 3A). However, analysis of all 272 miRNAs showed that only 15 (5.5%) in Met/Diest experienced a 1.5-fold increase compared to Pro/Est, while a 1.5-fold increased expression level in Pro/Est was seen for 19 (7.0%) of the miRNAs. Thus for the majority of the miRNAs, expression levels in OVS were unchanged between the two samples, as seen by the positive correlation in Fig. 3B.

### Validation of OVS miRNA abundance in Pro/Est and Met/Diest

To further investigate miRNAs in OVS, the expression levels of four selected miRNAs were quantified by RT-qPCR. Selection of the miRNAs for experimental validation of the next-generation sequencing data and for comparison of OVS and oviductal epithelia was based on their abundance during the estrous cycle, as well as their presence in sperm and in the murine epididymal epithelia where they are likely to be secreted in epididymosomes^24^. *MiR-143-3p* (assay ID 205992), was chosen because it is the most abundant in OVS in both estrous groupings (Supplementary Table S1), it is found predominantly in caput and corpus sperm, and is present in caudal epididymal epithelial cells^24^; *miR-let-7a-5p* (assay ID 205727) is found predominantly in caput sperm and is present in all three epididymal epithelial cell types^24^; *miR-22-3p (*assay ID 204606) is expressed at significantly higher levels in Met/Diest than in Pro/Est (Fig. 3A), is found predominantly in caudal sperm, and is present in the three epididymal epithelial cell types^24^; *miR-34c-5p* (assay ID 205659) is similar to *miR- 22-3p* in being predominant in caudal sperm and in all epididymal epithelia^24^, and was selected because it is required for the first zygotic cleavage division^25^.

**Figure 4:**
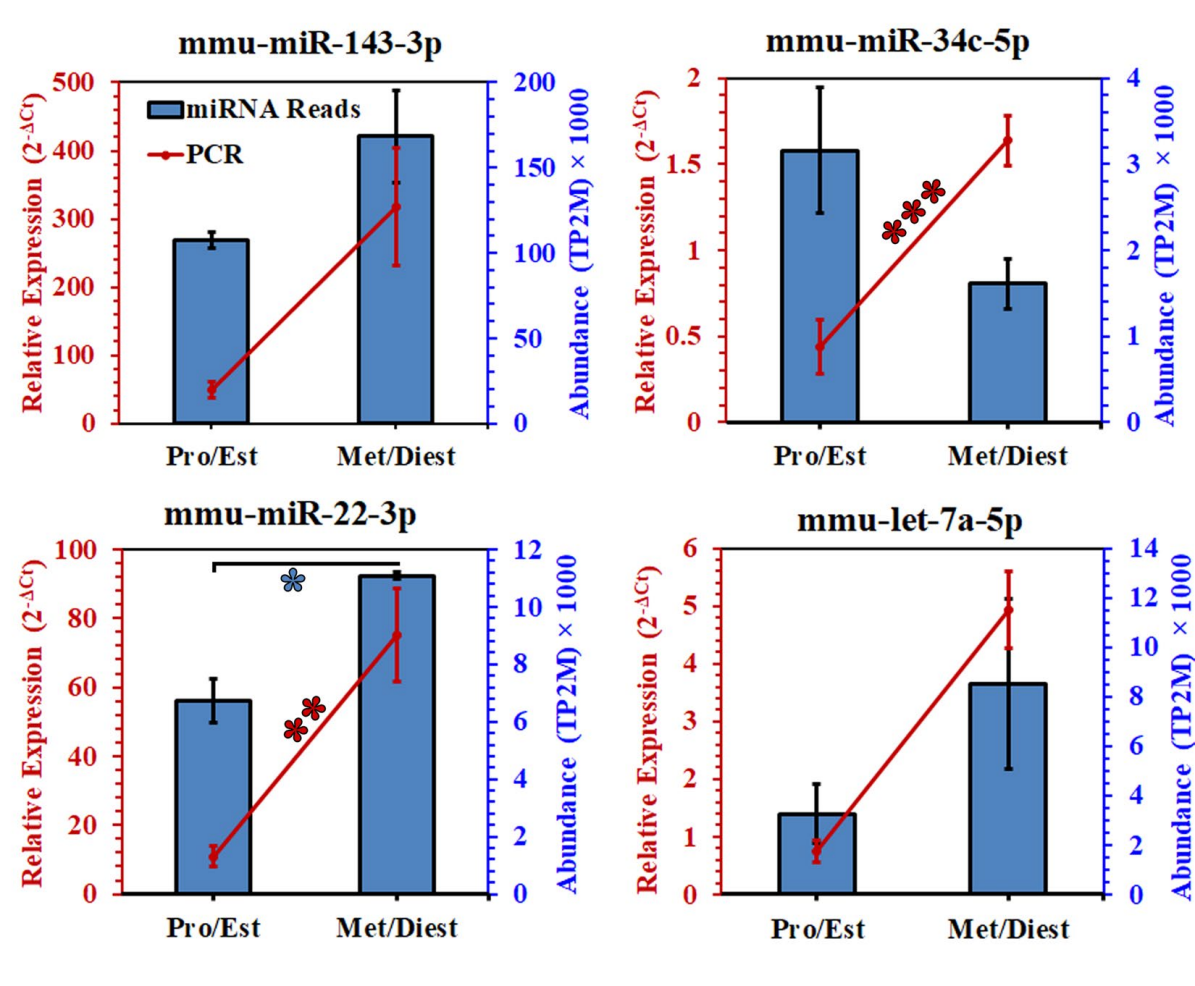
RT-qPCR validation of the abundance and differential expression of selected miRNAs in OVS from Pro/Est and Met/Diest. MiRNA abundance from sequencing is represented as blue columns while the relative abundance (2^−ΔCt^) of each miRNA assessed by RT-qPCR is represented by the red line graphs. For 3 of the 4 miRNAs (*miR-143*, *miR-22*, and *let-7a)*, expression levels were higher in Met/Diest for both the sequencing and RT-qPCR data, and for *miR-22* the differences were significant (^*^P < 0.05; ^**^P < 0.04). For *miR-34c*, only the RT-qPCR data showed a significantly (^***^P < 0.003) higher expression level at Met/Diest. U6 small nuclear RNA was used as an endogenous control to normalize expression levels of miRNAs from RT-qPCR.

The sequencing data for all assessed miRNAs from OVS isolated from the groups were confirmed by RT-qPCR quantification (Fig. 4). In general the trends of miRNA abundance within the next-generation sequencing and RT-qPCR data are similar in corresponding samples. For example, the relative abundance of the selected miRNAs, as detected by RT-qPCR, is in the same rank order as data obtained from the next-generation sequencing which is as follows: *miR-143-3p*, *miR-22-3p*, *let-7a-5p*, *miR-34c-5p* (Fig. 4, Supplementary Table S1). In the same context, the abundance of 3 of the 4 miRNAs; namely, *miR-22-3p*, *miR-143-3p* and *let-7a-5p* was higher in OVS isolated from Met/Diest than in Pro/Est by sequencing and RT-qPCR, both of which showed that in *miR-22-3p* the differences between the two groups were significant (*P < 0.05; **P < 0.04) (Fig. 4). While the abundance of *miR-34c-5p* was not significantly different between the two groups with sequencing, RT-qPCR showed it to be significantly higher in Met/Diest (***P < 0.003) than in Pro/Est (Fig. 4).

### MiRNA candidates are significantly more abundant in OVS than in parental oviductal cells

**Figure 5:**
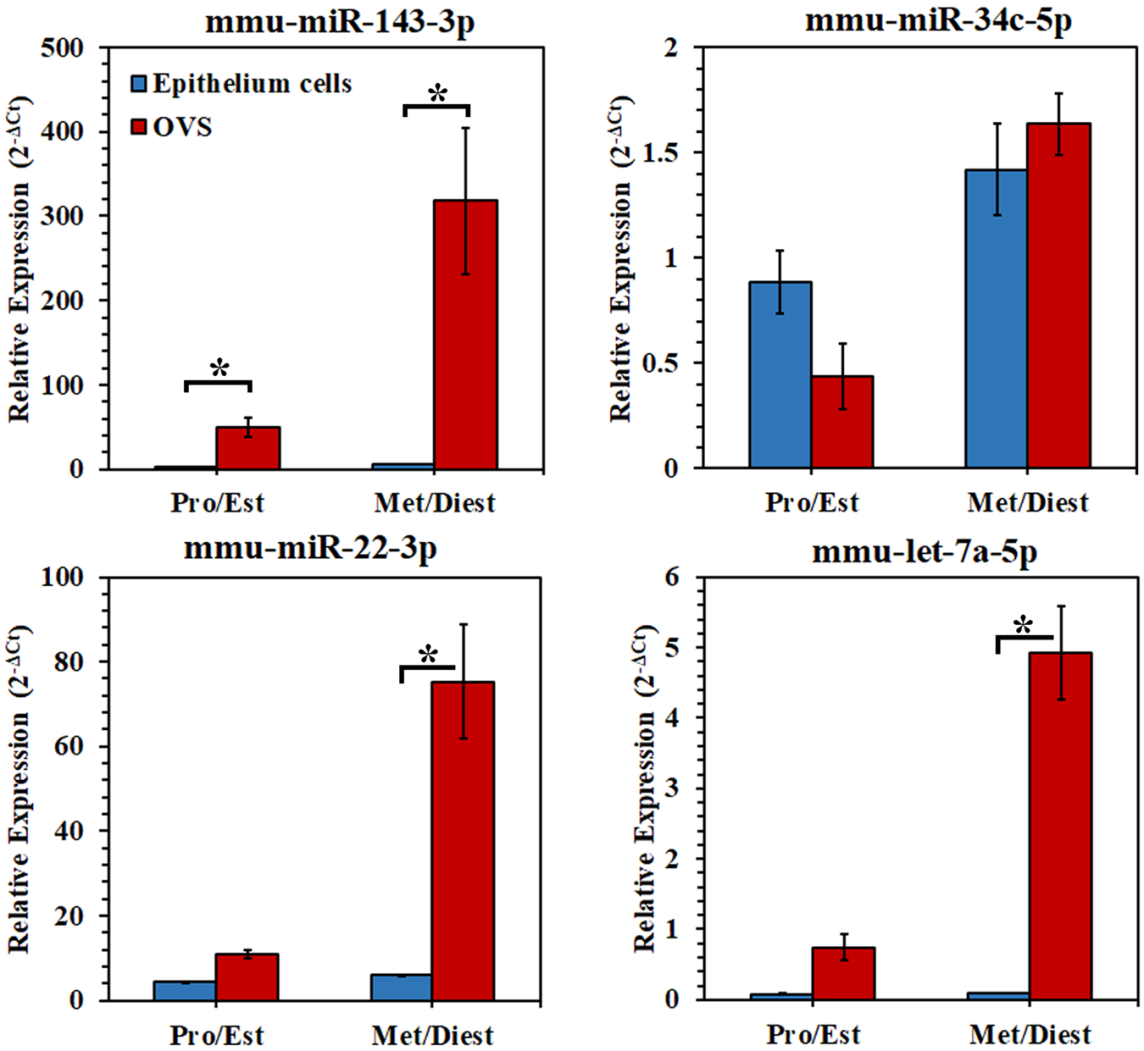
Comparison of the abundance of the selected miRNAs in oviductosomes and oviductal epithelium tissue, using RT-qPCR. (**A**) The presence of the four selected miRNAs (*miR-143*, *miR-34c*, *miR-22*, *let-7a*) in oviductosomes (red columns) was detected in oviductal epithelial tissue (blue columns) from Pro/Est and Met/Diest females, using RT-qPCR. For both OVS and epithelial cells, expression levels were higher in Met/Diest for all miRNAs except *let-7a* where similar levels were seen for Pro/Est and Met/Diest in oviductal cells. With paired *t*-tests, abundance was significantly higher (*P < 0.05) in OVS compared to oviductal cell in Met/Diest or in both groups for all miRNAs except *miR-34c-5p* where the levels were similar. RT-qPCR was analyzed in duplicate using pooled samples. Expression levels were normalized using U6 small nuclear RNA as an endogenous control.

To validate the presence of OVS miRNAs in oviductal epithelial cells, we quantified the levels of the four selected miRNAs (*miR-143-3p*, *miR-22-3p*, *let-7a-5p*, and *miR-34c-5p*) in the parental cells from which OVS are derived and in OVS. All four miRNAs detected in OVS are present in microdissected oviductal epithelial cells from the Pro/Est and Met/Diest groups (Fig. 5). In both OVS and the parental oviductal epithelial cells, 3 of the 4 candidate miRNAs were more abundant in Met/Diest than in Pro/Est. *Let-7a-5p* was the outlier, as the levels of expression in oviductal epithelial cells were similar for the two groups, unlike OVS (Fig. 5). Remarkably in OVS, abundancy of all miRNAs (except *miR-34c-5p)* was significantly (*P < 0.05) higher than in epithelial cells for Met/Diest or Met/Diest and Pro/Est, suggesting selective packaging. For *miR-34c-5p*, the expression levels in OVS are comparable with those in epithelial cells (Fig. 5).

### OVS are capable of delivering miRNAs to sperm *in vitro* following co-incubation

**Figure 6:**
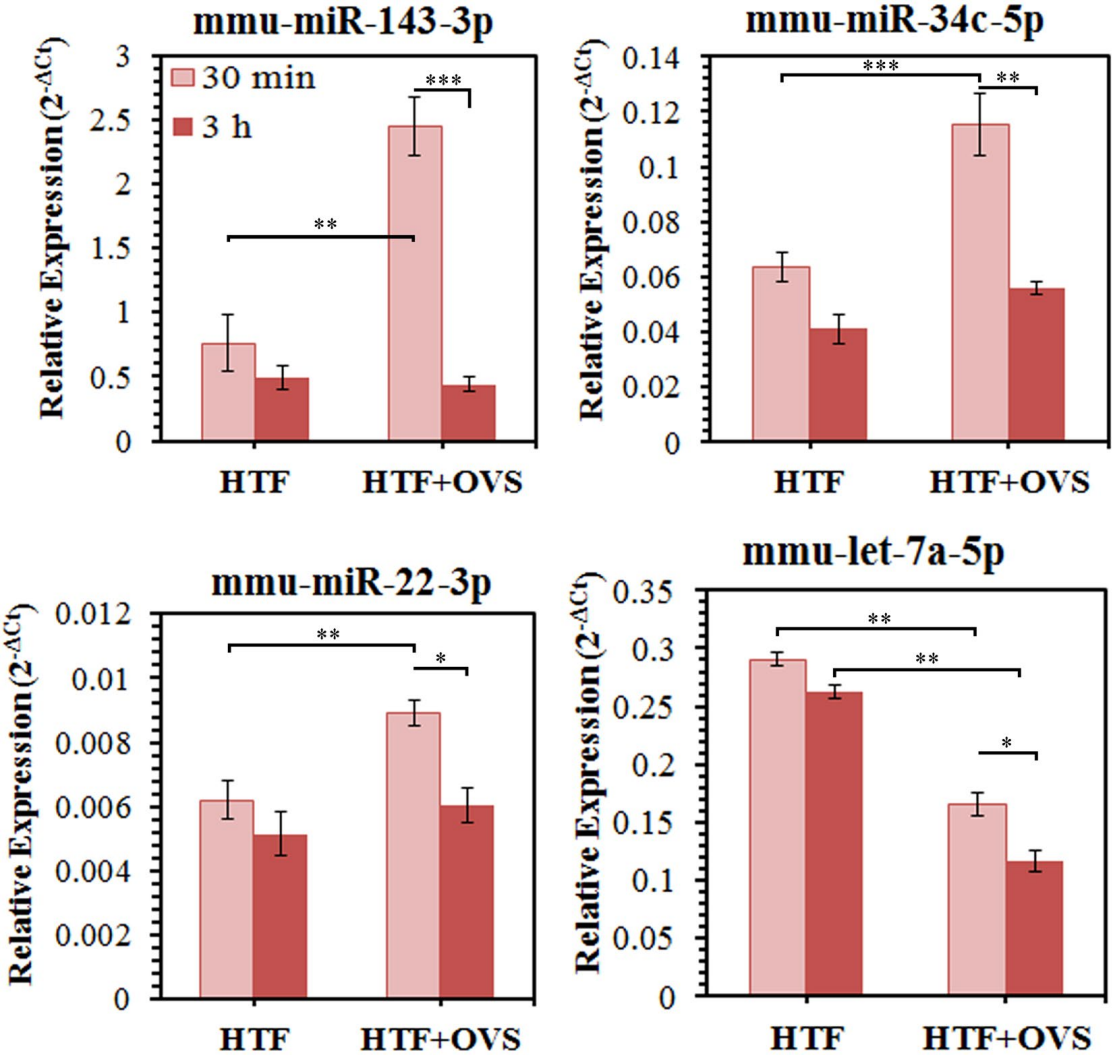
MiRNAs levels were significantly altered in sperm after OVS-sperm interaction, following co-incubation of sperm with estrus OVS. RT-qPCR reveals that for 3 miRNAs (miR-*143*, *miR-34c*, and *miR-22*) sperm show significantly elevated levels, reflecting oviductosomal delivery of miRNAs after co-incubation with OVS for 30 min only. Control sperm were co-incubated in the OVS vehicle, HTF. The fourth miRNA, *let-7a -5p*, showed significantly reduced levels in sperm after their co-incubation with OVS for 30 min and 3 h. RT-qPCR analyses were performed in triplicate, using pooled samples. Values are the average frequencies of miRNA for each sample. Significant levels are *P < 0.05, **P < 0.01, ***P < 0.001.

To determine if OVS are capable of delivering miRNAs to sperm, OVS-sperm interactions were fostered by co-incubation of sperm and OVS collected during estrus. The four miRNAs used for the validation of the sequencing data (*miR-143-3p*, *miR-22-3p*, *let-7a-5p*, *miR-34c-5p*) were selected to investigate the transfer of miRNA cargo to sperm. Figure 6 shows that at 30 min, 3 (*miR-143-3p*, *miR-22-3p*, *and miR-34c-5p*) of the 4 miRNAs had expression levels that were significantly (**P < 0.01 or ***P < 0.001) higher in sperm co-incubated in OVS than in HTF, the control vehicle. Only for *let-7a-5p*, the expression level was not elevated in sperm, but rather significantly (**P < 0.01) decreased, following OVS co-incubation. In general, the levels of the four miRNAs in sperm were higher after 30 min than after 3 h co-incubations for both sperm co-incubated in OVS and the HTF control (Fig. 6). This is likely a reflection of reduced sperm viability after 3 h compared to 30 min.

### Co-incubation of sperm with OVS loaded with labeled synthetic miRNAs reveals internalization of the labeled miRNAs and their localization to specific sperm head sub-compartments

**Figure 7:**
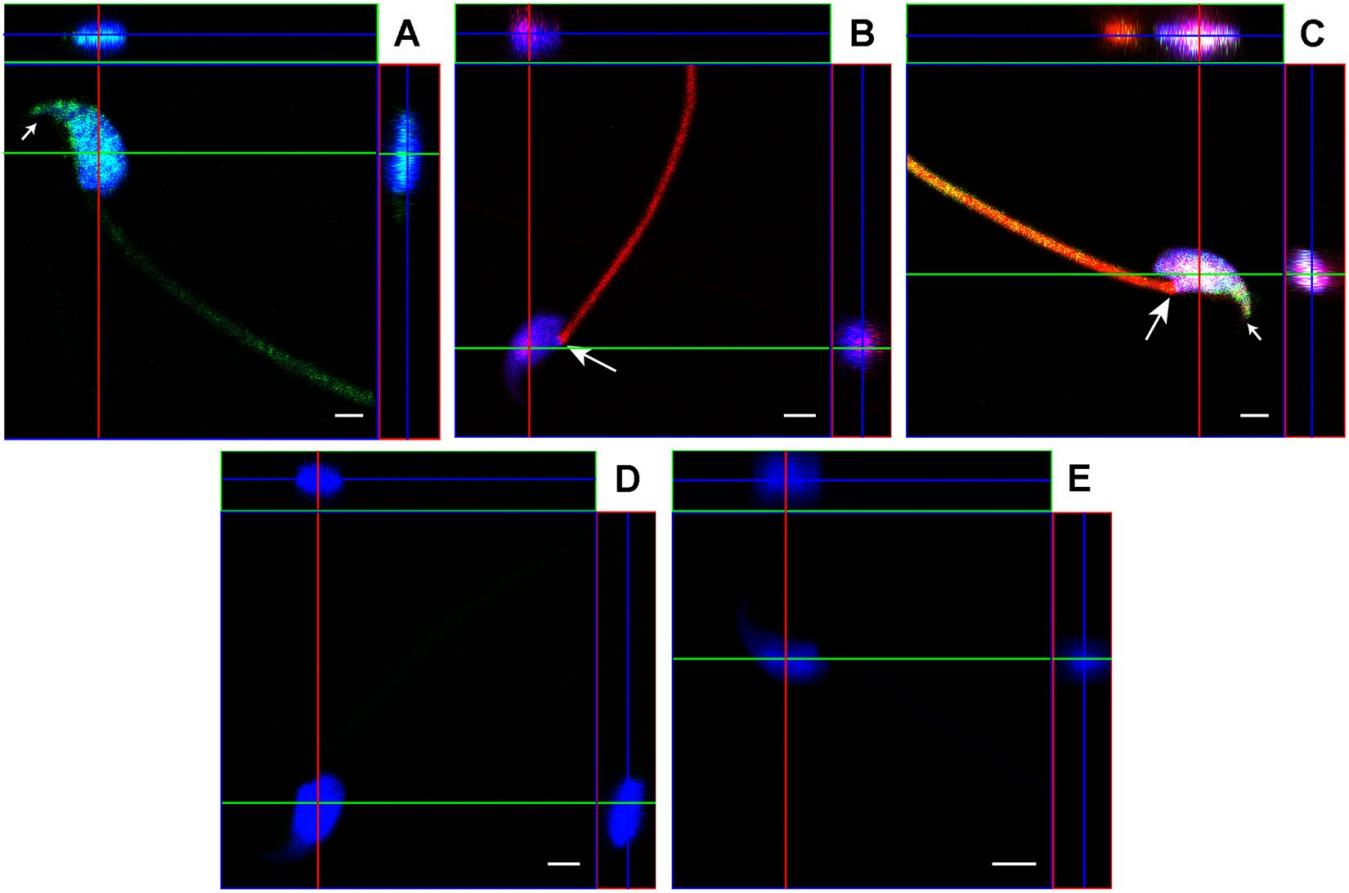
Co-incubation of sperm with OVS loaded with labeled miRNAs revealed the intracellular location of transferred miRNAs and the localization of individual miRNAs to specific sperm head sub-compartments. Following co-incubation of sperm and OVS loaded with fluorescently labeled synthetic (**A**) *miR-143-3p*, or (**B**) *miR-34c-5p*, or (**C**) both, sperm were visualized using confocal microscopy in an orthogonal projection composed of optical z-planes. Orthogonal projections of a sperm demonstrate a co-localization between *miR-143-3p* (green) and *miR-34c-5p* (red) with DAPI-stained nucleus (blue). Co-localization of all three (the green, the red, and the blue from the DAPI-stained nucleus) gives a white coloration (Fig. 7C). The large arrow shows the location of the centrosome with the high intensity red staining of *miR*- *34c-5p*, while the small arrow is over the tip of the acrosome where *miR-143-3p* is concentrated. Sperm co-incubated with the free *miR-34c-5p* mixture used for loading OVS (**D**) or with OVS that were not loaded with miRNAs (**E**) were used as negative controls. The green, red, and blue lines indicate the positions at which the orthogonal surfaces intersect. Scale bars = 2 µm.

To confirm that the significant increase in miRNA expression after co-incubation of sperm and OVS resulted from incorporation of the miRNAs in sperm and not merely OVS sticking to the sperm membrane, a minimum of 10 sperm were imaged after co-incubation with estrus OVS loaded with either labeled synthetic *miR-143-3p* or *34c-5p* or both. After processing, sperm were analyzed with confocal microscopy using Z-stack series in orthogonal representations. The label was seen scattered on the head and on the midpiece of the flagellum to varying degrees (Fig. 7**)**, in a pattern similar to that reported for labeled OVS detected by nanoscopy^22^. Interestingly, in addition to the scattered labeling (which overlapped in sperm co-incubated with OVS loaded with both labeled miRNAs), in 100% of the cells the red signal for *miR-34c-5p* was sequestered in or near the centrosome at a high intensity (Fig. 7B,C, Supplementary Fig. S2). The green label for *miR-143-3p* was seldom detected at this location but was seen concentrated at the tip of the acrosome (Fig. 7A,C, Supplementary Fig. S3). Control sperm incubated in the presence of free labeled *miR-143-3p* or *miR-34c-5p* (Fig. 7D) in the mixture used for loading the OVS, or in OVS without labeled miRNAs (Fig. 7E), showed no signal or autofluorescence. The absence of a signal from the free labeled miRNAs in the control (Fig. 7D) is consistent with the report that gametes are resistant to uptake of biomolecules^26^.

Z-stacks of confocal images of sperm co-incubated with OVS loaded with both labeled miRNAs revealed in orthogonal projections the spatial overlap between the blue DAPI stain of the sperm head and the transferred red and green label of the miRNAs (Fig. 7C). The presence of the red and/or green signals at the internal optical z-planes indicates that the labeled miRNAs are incorporated within the sperm. The spatial overlap between DAPI-stained nucleus and OVS-borne miRNAs was confirmed by three-dimensional reconstruction (Supplementary Fig. S3) using the orthogonal view of images obtained in the z plane.

### Target and enrichment analysis reveals that OVS miRNAs target genes involved in cellular growth and proliferation as well as embryonic development

We collected the experimentally validated targets for the top 25 differentially expressed miRNAs in Fig. 2 from miRTarbase release 7.0^27^. A total of 1296 miRNA-target pairs were collected from human (1071 pairs), mouse (194 pairs) and rat (31 pairs), corresponding to 752 unique EntreGene IDs and 669 unique genes. Since human contained the majority of annotations, we selected this organism as representative for further analysis. All the data were consolidated to produce a final list of 665 unique protein coding genes mapped to human UniProt accessions (Release 2018-01)^28^. This set was used for functional overrepresentation and enrichment analysis. As seen in Table 1, the miRNA target list contains several embryonic developmentally-related genes including *BCL2*, *CDK6* and *c-MYC*^25,29^.

**Table 1:** Gene targets which are regulated by 4 or more miRNAs from the top 25 miRNAs in Fig. 2.

Targets	Count of miRNAs	miRNA list
*C-KRAS*	**7**	***let-7a-5p***; *let-7g-5p*; ***miR-143-3p***; *miR-145a-5p*; *miR-181a-5p*; *miR-181c-5p*; *miR-340-5p*
*PTEN*	**8**	*miR-10a-5p*; *miR-10b-5p*; *miR-148a-3p*; *miR-181a-5p*; *miR-181c-5p*; *miR-21a-5p*; ***miR-22-3p***; *miR-26a-5p*
*AKT1*	**7**	*miR-10b-5p*; ***miR-143-3p***; *miR-192-5p*; *miR-196b-5p*; *miR-199a-3p*; ***miR-22-3p***; *miR-340-5p*
*BCL2*	**6**	***miR-143-3p***; *miR-148a-3p*; *miR-181a-5p*; *miR-181c-5p*; *miR-192-5p*; ***miR-34c-5p***
*CCND2*	**5**	***let-7a-5p***; *miR-145a-5p*; *miR-191-5p*; *miR-26a-5p*; *miR-340-5p*
*CDK6*	**5**	***let-7a-5p***; *miR-191-5p*; *miR-26a-5p*; *miR-340-5p*; ***miR-34c-5p***
*AGO1*	**5**	***let-7a-5p***; *let-7c-5p*; *let-7f-5p*; *let-7g-5p*; *let-7i-5p*
*HMGA2*	**4**	***let-7a-5p***; *let-7c-5p*; *let-7g-5p*; *miR-26a-5p*
*IL6*	**5**	***let-7a-5p***; *let-7c-5p*; *let-7f-5p*; *let-7i-5p*; *miR-26a-5p*
*JAG1*	**4**	***miR-143-3p***; *miR-199b-3p*; *miR-26a-5p*; ***miR-34c-5p***
*MET*	**5**	*miR-148a-3p*; *miR-199a-3p*; *miR-27b-3p*; *miR-340-5p*; ***miR-34c-5p***
*C-MYC*	**5**	***let-7a-5p***; *let-7c-5p*; *let-7g-5p*; *miR-26a-5p*; ***miR-34c-5p***
*STAT3*	**5**	***let-7a-5p***; *let-7c-5p*; *miR-148a-3p*; *miR-181a-5p*; *miR-340-5p*
*TGFBR1*	**5**	*let-7c-5p*; *let-7g-5p*; *miR-181a-5p*; *miR-181c-5p*; *miR-27b-3p*

**Figure 8:**
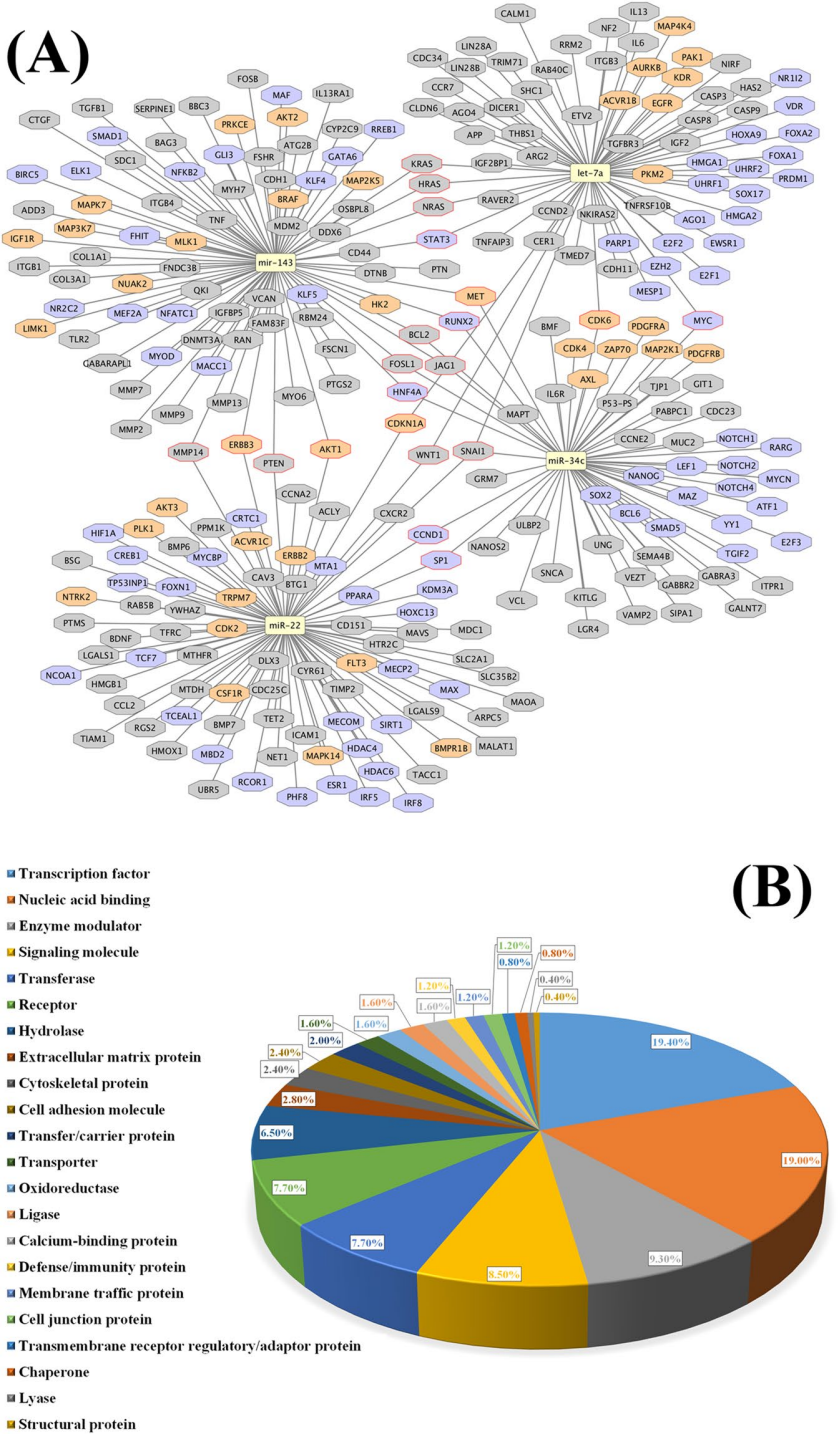
Targets for the four selected miRNAs include those for kinases and gene regulation and appear in a number of functional classes. (**A**) Cytoscape representation of miRNA-target network based on data and text mining. Targets containing UniProt keyword kinase or gene regulation are colored in orange or purple, respectively. MALAT1 is a noncoding RNA and is presented in a rectangle. Targets common to more than one miRNA are indicated with a red border. (**B**) Classification of targets in functional classes based on PANTHER classification.

**Figure 9:**
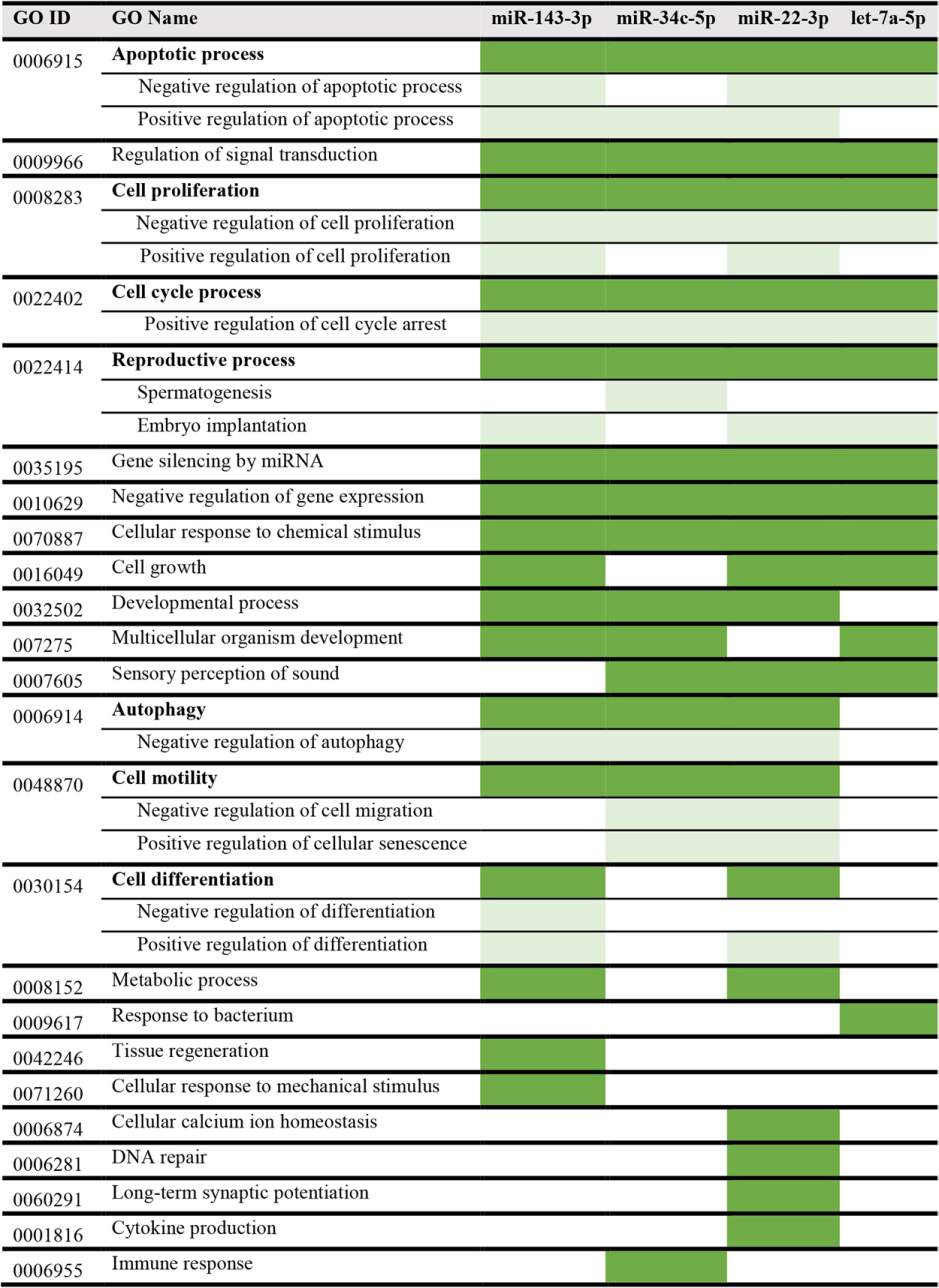
GO (gene ontology) annotation reveals that the 4 selected miRNAs used for validation of the sequencing data are associated with a variety of reproductive processes, among other processes. The dark green color indicates the processes mapped to a common high level GO hierarchy term, whereas the light green indicates the associated processes at a more specific level.

An analysis of the protein functions that are over-represented in the miRNA target list is seen in Table 2 which shows that the target list is mostly enriched in transcription factors, transcription regulators, and protein kinases. Analysis of enriched pathways from the target list was performed and is presented in Table 3. Prominent among the pathways are those associated with cellular growth and the cell cycle. We further extended the miRNA-target network using information extracted from the literature. Figure 8A shows the miRNA-network for the four selected miRNAs in Cytoscape, while Fig. 8B shows the functional classification of these targets. Nucleic acid binding proteins and transcription factors predominate in the targeted protein classes (Fig. 8B). Finally, using gene ontology (GO) annotations^30^ and information from the literature about the miRNAs, we recorded the processes in which the four selected miRNAs are involved. Figure 9 shows that these miRNAs are involved in the regulation of apoptosis, cell proliferation, cell cycle arrest and regulation, and signal transduction. Most importantly, there is evidence for the involvement of all four selected miRNAs in reproductive processes, three associated with embryo implantation, and the fourth, *miR-34c-5p*, with spermatogenesis and the early zygote (Fig. 9)^25,31^.

**Table 2:** Functional classification for the miRNA target list using PANTHER.

PANTHER Protein + A3:E21 Class	Count (total: 660)	Fold Enrichment	P-value	FDR
**Runt transcription factor**	3	31.88	5.23E-04	4.86E-03
▶Immunoglobulin fold transcription factor	6	6.17	8.04E-04	7.17E-03
▶Transcription factor	100	3.03	1.24E-21	2.65E-19
**TGF-beta receptor**	10	21.25	1.37E-09	3.25E-08
▶Cytokine receptor	13	2.59	2.50E-03	2.06E-02
▶Rreceptor	44	1.86	2.01E-04	2.26E-03
**Serine/Threonine protein kinase receptor**	10	19.93	2.16E-09	4.63E-08
▶Protein kinase	45	5.35	3.76E-18	2.68E-16
▶Kinase	48	4.02	7.11E-15	3.80E-13
▶Transferase	57	2.09	4.27E-07	8.30E-06
**C4 zinc finger nuclear receptor**	14	10.38	1.16E-09	3.11E-08
▶Zinc finger transcription factor	23	2.41	2.23E-04	2.38E-03
**Basic leucine zipper transcription factor**	7	7.97	7.46E-05	8.87E-04
**Non-receptor tyrosine protein kinase**	8	6.71	6.59E-05	8.29E-04
**HMG box transcription factor**	4	6.38	5.55E-03	4.09E-02
**Kinase activator**	11	6.15	6.01E-06	1.07E-04
▶Kinase modulator	15	3.8	2.67E-05	3.80E-04
▶Enzyme modulator	50	1.52	4.88E-03	3.73E-02
**Non-receptor serine/threonine protein kinase**	34	5.21	1.01E-13	4.34E-12
**Basic helix-loop-helix transcription factor**	12	4.84	1.99E-05	3.04E-04
**Homeodomain transcription factor**	12	3.64	2.36E-04	2.30E-03
▶Helix-turn-helix transcription factor	19	3.29	1.51E-05	2.49E-04
**Growth factor**	13	3.43	2.30E-04	2.34E-03
▶Signaling molecule	55	2.65	5.00E-10	1.53E-08
**Small GTPase**	8	3.11	6.10E-03	4.35E-02
**Annexin**	9	3.02	4.45E-03	3.53E-02
**mRNA processing factor**	12	2.94	1.35E-03	1.16E-02
▶Nucleic acid binding	104	2.11	2.05E-12	7.31E-11
**DNA binding protein**	30	2.29	6.24E-05	8.35E-04

The table includes the hierarchical relations between over-represented functional classes (indicated by arrows which point to the parent term). Columns are as follows: count indicating the number of proteins from a total of 660 that map to the given functional class, the fold enrichment indicates the fold of gene–term overrepresentation associated with a given gene list compared to a background list (human set), the corresponding P-value, and the False Discovery Rate (FDR) which is the expected proportion of the observed enrichments due to random chance.

**Table 3:** Enriched KEGG pathways obtained from the miRNA target list.

KEGG Term	Count (total:454)	Fold Enrichment	P-value	FDR
hsa04151:PI3K-Akt signaling pathway	81	3.57	3.60E-25	4.66E-22
hsa04010:MAPK signaling pathway	63	3.76	1.10E-20	1.42E-17
hsa04510:Focal adhesion	59	4.36	8.35E-23	1.08E-19
hsa04068:FoxO signaling pathway	52	5.91	6.02E-27	7.81E-24
hsa04014:Ras signaling pathway	48	3.23	4.82E-13	6.25E-10
hsa04550:Signaling pathways regulating pluripotency of stem cells	47	5.11	2.76E-21	3.58E-18
hsa04060:Cytokine-cytokine receptor interaction	43	2.85	7.02E-10	9.10E-07
hsa04015:Rap1 signaling pathway	42	3.04	1.33E-10	1.73E-07
hsa04390:Hippo signaling pathway	40	4.03	3.41E-14	4.43E-11
hsa04722:Neurotrophin signaling pathway	39	4.95	3.81E-17	4.93E-14
hsa04110:Cell cycle	38	4.66	9.85E-16	1.30E-12
hsa04380:Osteoclast differentiation	36	4.18	2.51E-13	3.26E-10
hsa04919:Thyroid hormone signaling pathway	35	4.67	1.59E-14	2.06E-11
hsa04810:Regulation of actin cytoskeleton	35	2.52	7.25E-07	9.39E-04
hsa04660:T cell receptor signaling pathway	34	5.02	3.83E-15	5.04E-12
hsa04668:TNF signaling pathway	33	4.74	6.66E-14	8.64E-11
hsa04066:HIF-1 signaling pathway	32	4.97	3.97E-14	5.14E-11
hsa04024:cAMP signaling pathway	32	2.46	4.23E-06	0.005
hsa04350:TGF-beta signaling pathway	31	5.62	2.52E-15	3.31E-12
hsa04012:ErbB signaling pathway	30	5.25	5.84E-14	7.57E-11
hsa04310:Wnt signaling pathway	30	3.31	1.34E-08	1.74E-05
hsa04062:Chemokine signaling pathway	29	2.37	2.62E-05	0.034
hsa04630:Jak-STAT signaling pathway	28	2.94	5.81E-07	7.53E-04
hsa04921:Oxytocin signaling pathway	28	2.70	3.31E-06	0.004
hsa04115:p53 signaling pathway	27	6.13	1.86E-14	2.42E-11
hsa04914:Progesterone-mediated oocyte maturation	26	4.55	1.30E-10	1.69E-07
hsa04915:Estrogen signaling pathway	26	4.00	2.62E-09	3.39E-06
hsa04910:Insulin signaling pathway	26	2.87	2.59E-06	0.003
hsa04620:Toll-like receptor signaling pathway	25	3.59	5.47E-08	7.10E-05
hsa04931:Insulin resistance	25	3.52	8.02E-08	1.04E-04
hsa04071:Sphingolipid signaling pathway	25	3.17	6.45E-07	8.37E-04
hsa04650:Natural killer cell mediated cytotoxicity	24	2.99	3.24E-06	0.004
hsa04917:Prolactin signaling pathway	23	4.93	3.44E-10	4.46E-07
hsa04912:GnRH signaling pathway	23	3.85	5.66E-08	7.33E-05
hsa04114:Oocyte meiosis	21	2.93	2.16E-05	0.028
hsa04210:Apoptosis	20	4.91	6.93E-09	8.99E-06
hsa04664:Fc epsilon RI signaling pathway	20	4.48	3.75E-08	4.86E-05
hsa04662:B cell receptor signaling pathway	20	4.41	4.86E-08	6.31E-05
hsa04520:Adherens junction	20	4.29	8.06E-08	1.05E-04
hsa04916:Melanogenesis	20	3.04	2.07E-05	0.027
hsa04150:mTOR signaling pathway	18	4.72	9.11E-08	1.18E-04
hsa04370:VEGF signaling pathway	17	4.24	1.16E-06	0.002
hsa04920:Adipocytokine signaling pathway	17	3.70	8.19E-06	0.011
hsa04666:Fc gamma R-mediated phagocytosis	17	3.08	8.98E-05	0.116
hsa04512:ECM-receptor interaction	17	2.97	1.39E-04	0.180
hsa04064:NF-kappa B signaling pathway	17	2.97	1.39E-04	0.180
hsa04720:Long-term potentiation	15	3.46	7.29E-05	0.094
hsa04320:Dorso-ventral axis formation	11	6.20	4.07E-06	0.005

Analysis was done using DAVID 6.8. Only non-disease pathways are shown. Columns are as follow: KEGG Term with the pathway ID and name, count indicating the number of genes from a total of 454 that map to the given pathway, the fold enrichment indicates the fold of gene–term overrepresentation associated with a given gene list compared to a background list (human set), the corresponding P-value, and the False Discovery Rate (FDR) which is the expected proportion of the observed enrichments due to random chance.

## Discussion

The present study is the first to report the capability of OVS to carry and transfer miRNAs to an important target cell, spermatozoon, extending current knowledge of their characterization. MiRNAs are evolutionarily conserved noncoding RNA molecules of ~22 nucleotides long and are major regulators of post-transcriptional gene expression^32^. Some miRNAs are secreted by donor cells in EVs where they are encapsulated, remain stable, and can be delivered to recipient cells to impact gene expression of the target cell. MiRNAs typically bind to the 3′UTR (untranslated region) of target mRNAs. It should be noted that a single miRNA can target many genes by inducing degradation of mRNAs or by inhibiting mRNA translation^33–36^. In this study, using next-generation sequencing with a cutoff of at least 2TP2M, OVS were shown to carry 272 miRNAs common to both Pro/Est and Met/Diest. For the top 50, significant differences in expression levels with at least a 1.5-fold change were seen for only 6 (12%) and 2 (4%) miRNAs in the Met/Diest and Pro/Est groups. When the entire 272 miRNAs were considered, increases reflecting a 1.5- fold change were seen in Met/Diest for only 15 (5.5%) and for Pro/Est for 19 (7.0%) miRNAs. Thus, throughout the estrous cycle expression levels did not appear to vary much for the vast majority of the miRNAs. It is unclear if the uncoupling of the stages into the four individual stages would have revealed greater variation of miRNA expression during the cycle.

RT-qPCR of selected miRNAs, in general, confirmed their presence and relative levels of expression in OVS, with the order of the abundance of the miRNAs being the same as that seen in the next-generation sequencing. For example, *miR-143-3p* which returned the highest read counts in the sequencing had the highest relative expression normalized to U6 endogenous control. More importantly, with both sequencing and RT-qPCR the trend for the relative levels of expression with respect to Pro/Est and Met/Diest was the same for 3 of the 4 miRNAs including *miR-22-3p* which had significantly higher levels in Met/Diest with both assays. Although we are unable to provide an explanation for the finding that *miR-34c-5p* did not trend with the majority with both assays, overall the qPCR observations provide validation for the presence and expression levels of the miRNAs.

Further validation of the miRNA expression in OVS was obtained from RT-qPCR of the oviductal epithelial cells, the parental cells from which OVS are derived. All miRNAs analyzed were shown to be present in the parental cells and, except for *let -7a- 5p*, existed in the same relative abundance as seen in OVS, with higher levels in Met/Diest than in Pro/Est. Abundance was unchanged throughout the estrous cycle for *let-7a-5p* in oviductal epithelial cells. However, *let-7a-5p* was among the majority of the miRNAs where transcripts were significantly more abundant in OVS than in parental cells, while *miR-34c-5p* was the outlier. This significant difference in miRNA abundance between parental cells and OVS is consistent with the finding that there is selective rather than stochastic packaging of cargo constituents in EVs, and consequently there is enrichment for both proteins and miRNAs in EVs^17,37,38^. To date, the exact mechanism(s) by which this occurs has not been unraveled. We also do not have an explanation for the differential behavior of *miR-34c-5p* with respect to a lack of difference in the abundance of transcript in OVS and parental cells.

Of the top 25 miRNAs, 16 (64%, including the most abundant miRNA, *miR-143-3p*), are also present in caudal mouse sperm, where the miRNA signature was shown to be substantially modified during epididymal maturation via sperm interaction with epididymosomes^19,24^. When OVS and sperm were co-incubated for 30 min, there were significant increases in the levels of three of the four miRNAs studied, indicating that OVS can transfer miRNAs to sperm similar to their transfer of proteins^3,5^. Our inability to detect increases after co-incubation for 3 h is highly likely due to reduced viability of the cells, since OVS only interact with live cells as shown in a FRAP (fluorescence recovery after photo-bleaching) assay^22^.

To ensure that the increased expression of the miRNAs after OVS-sperm interaction was a result of miRNA incorporated in sperm and not merely due to the presence of OVS adhering to the sperm surface (although the latter could not explain the significant decrease seen for *let 7a-5p*), we verified that synthetic labeled miRNAs loaded in OVS were intracellularly located in sperm after sperm-OVS co-incubation. Using confocal microscopy with z-stacking and 3D imaging, we showed incorporation of the labeled miRNAs within the sperm head. These results provide evidence that the endogenous OVS miRNAs are also incorporated within the sperm after delivery, and are consistent with the significant increases in the three miRNAs seen after sperm-OVS co-incubation.

Transfer of cargo constituents of OVS has been shown to occur via a fusogenic mechanism, involving integrin receptors on sperm and OVS^22^. Interestingly, one of the three miRNAs that was increased after co-incubation, *miR-34c-5p*, is known to initiate the first cleavage division in the mouse and provides the outstanding example of the impact of a single paternally-derived miRNA on embryonic development^39^. *MiR-34c-5p* which modulates *Bcl2* expression (Table 1) is found in comparable levels in zona-bound sperm and zygotes, but not in oocytes^25^. Therefore, zygotic *miR-34c-5p* is sperm-derived and it was shown that the presence of an inhibitor significantly suppressed the first division by blocking DNA synthesis^25^. Our findings indicate that the sperm’s endowment of *miR-34c-5p*, obtained in spermatogenesis and during epididymal maturation via epididymosomes^19,24^, is highly likely to be enhanced by sperm-OVS interaction in the oviduct just prior to fertilization.

The distinctly different localization of labeled *miR-34c-5p* and *miR-143-3p* in the sperm head compartments after uptake, not only provides evidence for the internalization of OVS miRNA cargo in sperm, but reveals that after delivery the miRNAs are sorted or sequestered to regions where they function physiologically. *MiR-34c-5p* was localized in the highest concentration at or near the centrosome which organizes microtubules that pull chromatids apart during cell division. This localization is relevant to *miR-34c-5p’s* role in promoting the first cleavage division^25^, and argues for the functional activity of OVS-borne miRNAs that are delivered to sperm. Our study highlights, for the first time, the role of OVS in this critical paternal contribution of miRNA to the zygote where its localization is consistent with its functional activity. The physiological significance of the high concentration of *miR-143-3p* seen over the tip of the acrosome (Fig. 7A,C, Supplementary Fig. S3) which undergoes exocytosis is unknown. Of great interest will be determining how miRNAs once delivered to the sperm find their way to specific head sub-compartments where they accumulate, to perform their function.

Since zygotic *miR-34c-5p* is sperm-derived, being highly expressed in spermatids, the acquisition of this miRNA from OVS in addition to that from epididymosomes may be a failsafe mechanism to ensure that sperm at the fertilization site have the required critical level of this miRNA. During epididymal maturation, *miR-34c-5p* is mainly acquired in the cauda^24^ which is the direct source of ejaculated sperm. It should be noted that ejaculated sperm are heterogeneous with respect to the length of time that they reside in the cauda prior to ejaculation. Sperm that arrived in the cauda just prior to ejaculation will have acquired much less *miR-34c-5p* and other miRNAs, via epididymosomes, than those residing for longer periods which could be up to 2 weeks in the mouse^40^. In the situation where ejaculation occurs soon after sperm arrival in the cauda, acquisition of additional *miR-34c-5p* via OVS may ensure that fertilizing sperm have the required critical level of this miRNA to initiate the first cleavage division.

The finding that one miRNA, *let-7a-5p*, did not increase in sperm after co-incubation, but rather significantly decreased is intriguing. It indicates that OVS not only can transfer miRNA from its cargo, but can impact sperm in a manner that reduces the level of an endogenous miRNA. Transfer of miRNAs to mouse sperm from EVs was recently shown *in vitro* for epididymosomes: all five candidate miRNAs (which did not include *let-7a-5p*) studied showed significant increases in sperm after sperm-epididymosome co-incubation^19^. Our results are the first finding of a decrease in a miRNA in sperm following their co-incubation with EVs.

This decrease in sperm miRNA induced by sperm-OVS interaction may be reflecting an unconventional characteristic of OVS, which are heterogeneous in nature^22^, or of *let-7a-5p*, or of both. Notably, among the most abundantly expressed OVS miRNAs are members of the *let 7* family, five (*let 7f-5p*, *let 7c-5p*, *let 7i- 5p*, *let 7a-5p*, *let 7g- 5p*, in order of decreasing abundance) of which are in the top 25 detected (Fig. 2). Interestingly, all 5 family members are abundantly expressed in caput mouse sperm and undergo drastic reductions in abundance in corpus and caudal sperm during the modifications that accompany epididymal maturation^24^. These modifications involve both loss and gain of miRNAs and while the mechanism responsible for the gain has been identified as epididymosomal transfer, the mechanism underpinning the loss is unknown, although miRNA elimination in cytoplasmic droplets has been suggested^24^. The latter is unlikely in our system as with the loss of *let 7a-5p* we detected no significant difference in the frequency of sperm which retained cytoplasmic droplets, after co-incubation with OVS compared to the carrier control (Supplementary Table S2). Our finding of a decrease in *let-7a -5p* with sperm-OVS interaction *in vitro* mimics the decrease seen physiologically in caudal sperm^24^ and suggests that during epididymal maturation the reduction of *let-7a-5p* and other miRNAs, similar to an increase in abundancy of miRNAs, may be a result of sperm-epididymosome interaction. Whether or not EVs introduce molecules that degrade *let-7a-5p* or are able to induce sperm to release this miRNA, remains to be determined. It should be noted that it has been reported that dying neurons release *let-7b in vitro*^41^.

*Let -7a- 5p*, along with family members, is known to play a major role in development^42,43^, and Table 1 shows that it targets several embryonic developmentally-related genes including *CDK 6* and *c-MYC*^29^. In general, analysis of all the targets of the top 25 miRNAs reveals a preponderance of transcription factors, transcription regulators as well as kinases (Table 2), with targeted pathways that include MAPK, P13K-AKT, and TGFB (Table 3). As these pathways regulate genes involved in early embryonic development and are centered on cellular growth and cell division, oviductosomal delivery of the miRNAs to the early embryo (which resides in the oviduct until the blastocyst stage) would be of physiological relevance. In this regard EVs from bovine oviductal epithelial cultured cells have been shown to have a positive effect on the quality of *in vitro* produced bovine embryos, suggesting a functional communication between the oviduct and the embryo during the early developmental stages^6,7^. It is of significance that all four selected candidate miRNAs are involved in processes that include cell proliferation, apoptosis, and the cell cycle (Fig. 9), all of which are critical for early embryonic development. Facilitated by removal of the zona or hatching of the blastocyst, OVS could deliver miRNAs to blastomeres to impact these processes. However, they could impact the morula or earlier stages, as EVs have been known to penetrate the zona and be internalized into blastomeres to impact gene expression^7,42^. In this vein, Alminana *et al*. have shown that bovine OVS mediate communication between the oviduct and the oocytes by delivering to the latter a variety of proteins^7^. Delivery of these proteins is likely accompanied by transfer of miRNAs that could impact the final stages of oocyte maturation and fertilization.

In addition to regulating genes in the gametes, the zygote, and the early embryonic stages, miRNAs in OVS could also impact gene expression in the endometrial lining of the uterus. As EVs are known to be involved in intercellular communication of both short- and longer-range signaling events^44–46^, OVS could travel to the cycling uterus and deliver miRNAs to the endometrial epithelial cells to induce physiological changes that impact the subsequent stage. Importantly, miRNAs transferred to the endometrial epithelium in the pregnant female could play a role in implantation, since 3 of the 4 candidate miRNAs in Fig. 9 are involved in embryo implantation^31^. Signaling from these miRNAs could contribute to the reported role of endometrial EVs in promoting embryo implantation and endometrial receptivity in the embryo-maternal cross-talk^16,47^. In this regard, bovine oviductal EVs were shown to increase blastocyst rate and to extend embryo survival^7^, while EVs released from bovine oviductal cultured cells had a positive effect on embryo quality of *in vitro* produced embryos^6^.

## Conclusions

MiRNAs in OVS, detected for the first time, have similar expression levels for the majority throughout the murine estrous cycle where they target genes encoding a variety of proteins. The functions of these proteins are centered on cell division and cellular growth, and consequently these proteins are involved in pathways that promote embryonic development. The majority of the top 25 miRNAs are expressed in mature sperm and our results reveal that co-incubation of OVS with sperm can lead to both a gain and a loss of specific miRNAs in sperm. As this mimics what occurs physiologically during epididymal sperm maturation, our findings suggest the involvement of epididymosomes in the loss of miRNAs in maturing sperm. Importantly, our results indicate that miRNAs delivered by OVS to sperm are sorted to specific head sub-compartments to perform their functional activity, as seen for *miR-34c-5p* at the centrosome. In addition to miRNA transfer to sperm there is the potential transfer of OVS miRNAs to blastomeres in the developing embryo, to potentially improve embryo quality. Based on miRNAs target enrichment, the potential transfer of oviductosomal miRNAs to the endometrial epithelium could facilitate implantation and endometrial receptivity. Further study of oviductosomal miRNAs in the cross-talk between the embryo and the oviduct could provide knowledge and tools to improve the blastocyst rate along with blastomere number, blastocyst survival, and development in natural pregnancies, as well as those achieved using assisted reproductive technologies.

## Methods and Materials

### Mice and reagents

In this study, sexually mature 4–12 week-old females and 10–12 week-old male mice (FVB/N strain; Harlan, Indianapolis, IN) were used. The studies were approved by the Institutional Animal Care and Use Committee at the University of Delaware and were in agreement with the Guide for the Care and Use of Laboratory Animals published by the National Research Council of the National Academies, 8th ed., Washington, D.C. (publication 85–23, revised 2011). All enzymes and chemicals were purchased from Fisher Scientific Co. (Malvern, PA), Sigma (St Louis, MO) or Thermofisher (Carlsbad, CA), unless otherwise specified.

### Antibodies

Mouse monoclonal anti-CD9 antibody (SC-18869) was purchased from Santa Cruz Biotechnology (Dallas, TX). Secondary antibodies were purchased from Thermofisher. These antibodies were used in Western blot and immunoelectron microscopy.

### Superovulated females

In order to induce female mice into estrus, sequential intraperitoneal injections of pregnant mare serum gonadotropin (PMSG) (7.5 i.u.) and human chorionic gonadotropin (HCG) (7.5 i.u.) (Sigma-Aldrich, St. Louis, MO) were given 48 h apart. Females were sacrificed 13.5–14 h after the second injection, and the oviducts removed to isolate OVS or to embed in OCT (optimal cutting temperature) medium (Tissue Tek, Torrance, CA) for sectioning.

### Isolation of Oviductosomes (OVS)

At least 40 females provided oviductal tissues for purifying OVS for each of the two groups. Briefly, the tissues were minced in PBS and after gravity settling, the fluid was clarified by centrifugation (16,000 × g for 20 min) to eliminate cell debris, and tissue fragments. As described previously^3,48^, the supernatant is referred to as the oviductal luminal fluid (OLF). To isolate OVS, Pro/Est and Met/Diest OLFs were subjected to ultracentrifugation at 120,000 × *g* at 4 °C for 2 h, using a Beckman Optima 2–70 k ultracentrifuge and a Ti60 rotor, as described^48^. Following this the pellet, OVS, was re-suspended in PBS and stored at −80 °C until ready for processing (Fig. 1A).

### Collection of luminal fluids and tissue in naturally cycling females

Virgin females were categorized by the stages of estrous cycle based on the proportion of different cell types observed in the vaginal secretion^49^. After sacrifice, oviductal tissues were collected from females at each of the four stages: proestrus, estrus, metestrus, and diestrus and washed with PBS (estrus is the only stage in which females will mate). They were then embedded in OCT medium, and stored at −80 °C until sections were made. To collect oviductal luminal fluid to isolate OVS, tissues from the four stages of estrous cycle were combined into 2 groups: Pro/Est and Met/Diest, as previously described^3,5^.

### SDS-PAGE and western blot analysis

Extraction of proteins from OVS, collected in different stages of estrus, and epididymosomes (EPS) which were used as a positive control was performed as described previously^50^. The bicinchoninic acid protein assay Kit (Pierce, Biotechnology, IL, USA) was used for determination of total protein concentration in the lysates according to the manufacturer’s protocol. In order to prepare samples for electrophoresis, non-reducing conditions was used as described^3^. Briefly, samples were diluted in 2x Laemmli sample buffer with DTT (final concentration 100 mM) and urea (125 mg/ml) and incubated for 10 min at 37 °C. Twenty to 60 μg of proteins from tissues and fluids, respectively, were loaded per lane on 10% polyacrylamide gels and transferred onto a nitrocellulose membrane (Amersham Biosciences, UK). Blots were blocked for 1 h at RT and incubated in anti-CD9 (1:1000) primary antibody overnight at 4 °C, and processed as previously described^3,5^.

### Negative Staining for Transmission electron microscopy (TEM)

Nickel TEM grids (Electron Microscopy Sciences, Hatfield, PA, USA), 400 mesh with a formvar/carbon film, were floated on a drop of the fractions of purified OLF pellet suspension. The grids were then washed with several drops of water and then stained with 1% uranyl acetate, a phospholipid stain, before being subjected to microscopic analysis. Membrane vesicles were imaged using TEM (Zeiss LIBRA 120, Germany). Immunogold labeling was performed as previously described using anti-CD9 primary antibodies^3^.

### RNA Extraction and next-generation sequencing of the small RNA fraction

Oviductosomal miRNAs were extracted and purified using the Total Exosome RNA and Protein Isolation kit (Invitrogen #4478545) according to the manufacturer’s recommendations. The quality of the RNA samples was checked on the fragment analyzer to ensure that bands from contaminating cellular RNA were absent. Two biological replicates of small RNA libraries were made using the TruSeq Small RNA kit (Illumina) with 2 µg total RNA, and submitted for deep sequencing (combined depth of over 82 million reads) at the Delaware Biotechnology Institute Sequencing Core. Adaptor sequences were trimmed, sequences matching the genome more than 10 times and simple sequence repeats removed, and sequences 18–28 nt were matched to the pre-build mouse genome NCBIM37 bowtie 1 index with 0 mismatches allowed. MiRNAs were identified by exact match to annotated mmu-miRNAs in miRBase V19. Analysis was restricted to miRNAs present in at least 2TP2M in both biological replicates in either the Pro/Est or Met/Diest sample.

### Laser Microdissection (LM) of oviductal epithelia

To confirm and validate the data obtained from next-generation sequencing of miRNA in OVS, miRNA expression in the oviductal epithelia from females in different stages of the estrous cycle was analyzed. This was performed with the use of frozen (in OCT, as described above) oviductal tissue sections, 20 µm in thickness. Microdissection was performed as previously described^51^. Briefly, the Zeiss Laser Microdissection (PALM Microlaser Technologies AG, CombiSystem,Carl Zeiss, Germany) system was used for dissection of selected oviduct epithelial regions on the sections. Prior to microdissectioning, the sections were dehydrated by soaking the slides in 100% ethanol for 2 min followed by air drying for 5 min. After air drying, the target area, oviductal epithelial cells, was carefully laser-microdissected (LM) and placed in the lysis buffer from the RNA isolation kit. The tissues were captured under the following LM conditions: laser energy 100, laser focus 80–85, and cut speed 15. The microdissected total areas for each sample were approximately 400,000–550,000 μm^2^ (Supplementary Fig. S4). The microdissected samples were kept at −80 °C until RNA extraction.

### RNA extraction from Laser-microdissected (LM) oviductal epithelial cells and Reverse Transcription- Quantitative Polymerase Chain Reaction (RT-qPCR)

RNA was extracted from LM samples using the miRCURY™ RNA Isolation Kit (#300110, Exiqon Inc., MA, USA), according to the manufacturer’s protocol. Briefly, the tissues were lysed in 100 μl lysis buffer after vigorously vortexing for 5 min. An equal volume of 70% ethanol was added to the lysate and the sample vortex for 1 min, after which a column was assembled to bind miRNA. Using the wash solution, the column was washed 6 times to minimize the amount of genomic DNA contamination. Finally, the RNA was eluted from the column using 20 μM of elution buffer. The RNA concentrations were determined using the NanoDrop^®^ ND 1000 Spectrophotometer and then stored at −80 °C for further study.

Reverse transcription-quantitative PCR (RT-qPCR) of RNA from Pro/Est and Met/Diest oviductal epithelia and from OVS samples in triplicate were performed using the miRCURY LNA™ Universal RT microRNA PCR (Exiqon In., MA, USA), according to the manufacturer’s instructions. Briefly, total RNA (50–100 ng) from each sample was reverse transcribed at 42 °C for 1 h followed by inactivation at 94 °C for 5 min. The resulting cDNAs were diluted 1:80 and 1:40 for OVS and tissue, respectively. The conditions for miRNA amplification for each primer PCR cycle consisted of an initial denaturing step at 95 °C for 2 min; 40 cycles of denaturation for 10 s at 95 °C, annealing for 60 s at 56 °C. One microliter of ROX Passive Reference Dye (50 nM) (Invitrogen, 75768500UL) was added to the real-time PCR master mix. A reaction volume of 10 μl was applied for each sample. The real-time PCR panel was run on QuantStudio 6 Flex Real-Time PCR System (Applied Biosystems). MiRNAs for experimental validation of OVS and for comparison of OVS and oviductal epithelia were selected based on their abundance and differential expression during the estrous cycle, as well as their presence in sperm and in the epididymal epithelia. The primers used for the detection of expression of these miRNAs were purchased from Exiqon Inc. (MA, USA) and are listed in Supplementary Table S2.

Four endogenous controls (*U6 snRNA*, *Rnu1a1*, *Rnu5g*, and *Gapdh*), purchased from Integrated DNA Technologies (IDT) (Coralville, IA, USA) were selected for each sample and are listed in Supplementary Table S3. The results are provided in Supplementary Fig. S5. All samples were normalized using *U6 snRNA* as a positive control. The fold increase relative to control samples was determined by the 2^−ΔΔCt^ method. Thermal cycling conditions were as follows: 40 amplification cycles at 95 °C for 10 s, 60 °C for 1 min with 1.6 °C/s ramp-rate. The RT-qPCR products were loaded on 2% agarose gel stained with ethidium bromide.

### Transfer of OVS miRNAs to sperm following Sperm-OVS co-incubation

Cauda epididymis and vas deferens of 10 sexually mature males were minced in PBS with protease inhibitor at 37 °C and left for 10 min to allow sperm to swim out, as previously performed^3,5^. Tissue fragments were separated by gravity settling, and sperm were collected from the supernatant by centrifugation at 500 × g for 15 min. Following this, sperm were re-suspended in Human Tubal Fluid, HTF, sperm wash # 2005 (Invitrocare, Frederick, MD) and split into four aliquots. Each aliquot was incubated in 100 μl of HTF (control) or 100 μl OVS re-suspended in HTF (0.55ug/ul). The OVS were collected from females hormonally-induced into estrus. Sperm and HTF/OVS were co-incubated at 37 °C for 30 min or 3 h. After co-incubation, sperm were collected from the suspensions by centrifugation (500 × g for 15 min) and washed three times using PBS to remove any residual OVS. Sperm were then stored at −80 °C until used for RNA extraction. Following RNA extraction, the relative abundance of candidate miRNAs was measured by RT-qPCR in test and control samples to investigate miRNA delivery to sperm by OVS.

### OVS loading with fluorescently labeled synthetic miRNAs and confocal microscopic analysis of OVS miRNA transfer into sperm

Estrus OVS isolated from OLFs were loaded with synthetic *miR-34c-5p* and/or *miR-143-3p* conjugated with 5′ Atto 550 and 5′ Alexa 488 (IDT, Coralville, IA) respectively, using Lipofectamine**™** 3000 (Invitrogen, Carlsbad, CA). Briefly, OVS were diluted in RNase-free PBS in 1: 2.5-3 ratio and synthetic miRNA was added at a final amount of 10 pmol (Mix A). In a separate tube, Lipofectamine**™** 3000 was diluted with RNase-free PBS in 1:15 ratio (Mix B). After incubating at room temperature for 5 min, Mix A was transferred into Mix B in 1:1 ratio. Finally, the mixtures were incubated for 20 min at room temperature with intermittent mixing. The loading efficiency of miRNA into OVS was determined by observing microscopically the fluorescence intensity of the loaded OVS. Once OVS loading with the synthetic miRNAs was confirmed, sperm were co-incubated with the loaded OVS in the presence of HTF. After incubation with HFT/OVS for 30 min, sperm were collected by centrifugation and washed three times using PBS to remove any residual OVS and free miRNAs. Sperm were then fixed in 4% paraformaldehyde solution and mounted in a glass bottom petri dish. Fluorescence confocal images were captured using appropriate filter sets on an LSM 880 confocal microscope with AiryScan (Zeiss) fitted with a 63x objective. Comparison of the intracellular fluorescent signals of sperm (both by 3D imaging and optical sectioning with a pinhole diameter of 1 Airy Unit) from the experimental groups (sperm co-incubated with loaded OVS/HTF) and those from the control (sperm co-incubated with HTF containing the free miRNA/miRNAs mixture used for loading the OVS or with OVS without labeled miRNA) enabled us to determine that synthetic miRNAs loaded into OVSs were transferred into sperm in which they were incorporated in the head.

### Bioinformatics analysis of miRNA targets

Validated targets for the top 25 miRNAs were obtained from miRTarbase release 7.0^27^. Interactions with Support Type label “Weak” were not included. Interactions were collected for human, mouse and rat miRNAs. Mouse and rat targets were mapped to the corresponding human counterpart, and Protein Ontology was used to assist in the orthology mapping via SPAROL query^30^. The miRNA target gene list analysis was performed in the AmiGO 2 website (http://amigo.geneontology.org/amigo) running the PANTHER protein classification and statistical overrepresentation test (Released 2017-12-05), with the following settings: annotation version and release date: PANTHER version 13.0 Released 2017-11-12; reference list: *Homo sapiens* (all genes in database); annotation data set: PANTHER Protein Classification; and test type: Fisher’s Exact with FDR (false discovery rate) multiple test correction. DAVID Bioinformatics Resource (version 6.8)^52,53^ was used for KEGG pathway analysis. Annotations on selected miRNAs were collected directly from the AmiGO website (accessed on 2017-12-27) and from the literature using in-house developed text mining methods for extracting target and process information involving one or more of the selected miRNAs^54^. Cytoscape 3.6.0^55^ was used for building the miRNA-target network. The biological processes extracted via text mining were mapped to GO terms (semi-automatically) for integration of results with GO annotations. Finally, for visualization and comparison of common/related GO process terms among a selected set of miRNAs, GOView (http://www.webgestalt.org/GOView/) was used.

### Statistical analysis

Experiments were performed in triplicate and all data are expressed as means (±SEM).  The Student T-test and Chi-squared (χ^2^) analysis with Yates correction were used to determine statistical significance. Differences were considered to be significant when P < 0.05.

## Electronic supplementary material

Supplementary Information

Video S3a. Videos showing the internalization of the labeled miRNAs in 3D reconstructions

Fig. S3 Videos showing the internalization of the labeled miRNAs in 3D reconstructions
