# Supplementary material for: Murine Oviductosomes (OVS) microRNA profiling during the estrous cycle: Delivery of OVS-borne microRNAs to sperm where miR-34c-5p localizes at the centrosome
Source: Sci Rep. 2018 Oct 31;8:16094. doi: 10.1038/s41598-018-34409-4 (PMC6208369; doi:10.1038/s41598-018-34409-4)
Supplement: Supplementary file 1 — Supplementary Information [file 41598_2018_34409_MOESM1_ESM.pdf]

**Manuscript Title:** **Murine Oviductosomes (OVS) microRNA profiling during the estrous cycle: Delivery of OVS-borne microRNAs to sperm where *miR-34c-5p* localizes at the centrosome**

Zeinab Fereshteh[1], Skye A. Schmidt[2], Amal A. Al-Dossary[1,3], Monica Accerbi[2], Cecilia Arighi[4], Julie Cowart[4], Jia L. Song[1], Pamela J. Green[2], Kyungmin Choi[5] Soonmoon Yoo[5] and Patricia A. Martin-DeLeon[1]

[1]Department of Biological Sciences, University of Delaware, Newark, DE 19716, USA
[2] Department of Plant and Soil Sciences, Delaware Biotechnology Institute, University of Delaware, Newark, DE 19711, USA
[3]Present Address: Department of Biology, College of Medicine, Imam Abdulrahman Bin Faisal University, P.O. Box 1982, Dammam 31441, Saudi Arabia
[4]Center for Bioinformatics and Computational Biology, University of Delaware, Newark, DE 19711, USA
[5]A.I. DuPont Hospital for Children, 1600 Rockland Rd, Wilmington, Delaware 19803, USA

- Corresponding author: 219 McKinly Lab, Univ. Delaware, Newark, DE 19716, USA, Tel: 302- 831-2249: Fax: 302-831-2281, pdeleon@udel.edu
-

Running Title: The microRNA repertoire of oviductosomes includes microRNAs present in sperm

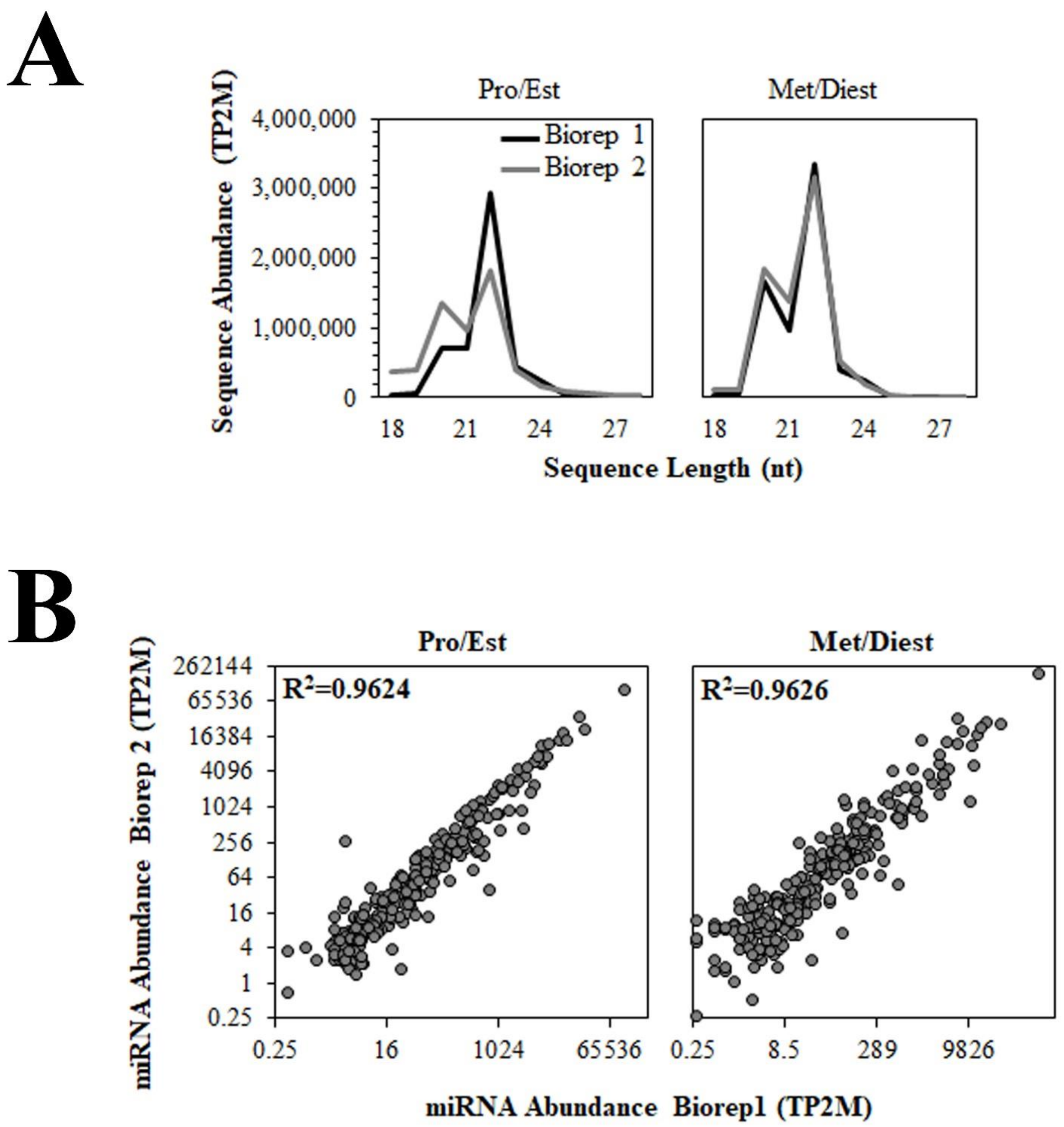


**Fig. S1.** Evaluation of the sequencing data after normalization shows good agreement between biological replicates for **A)** the sequence size profile and **B)** miRNA sequence abundance, for Pro/Est and Met/Diest samples. A strong positive correlation is shown in B.

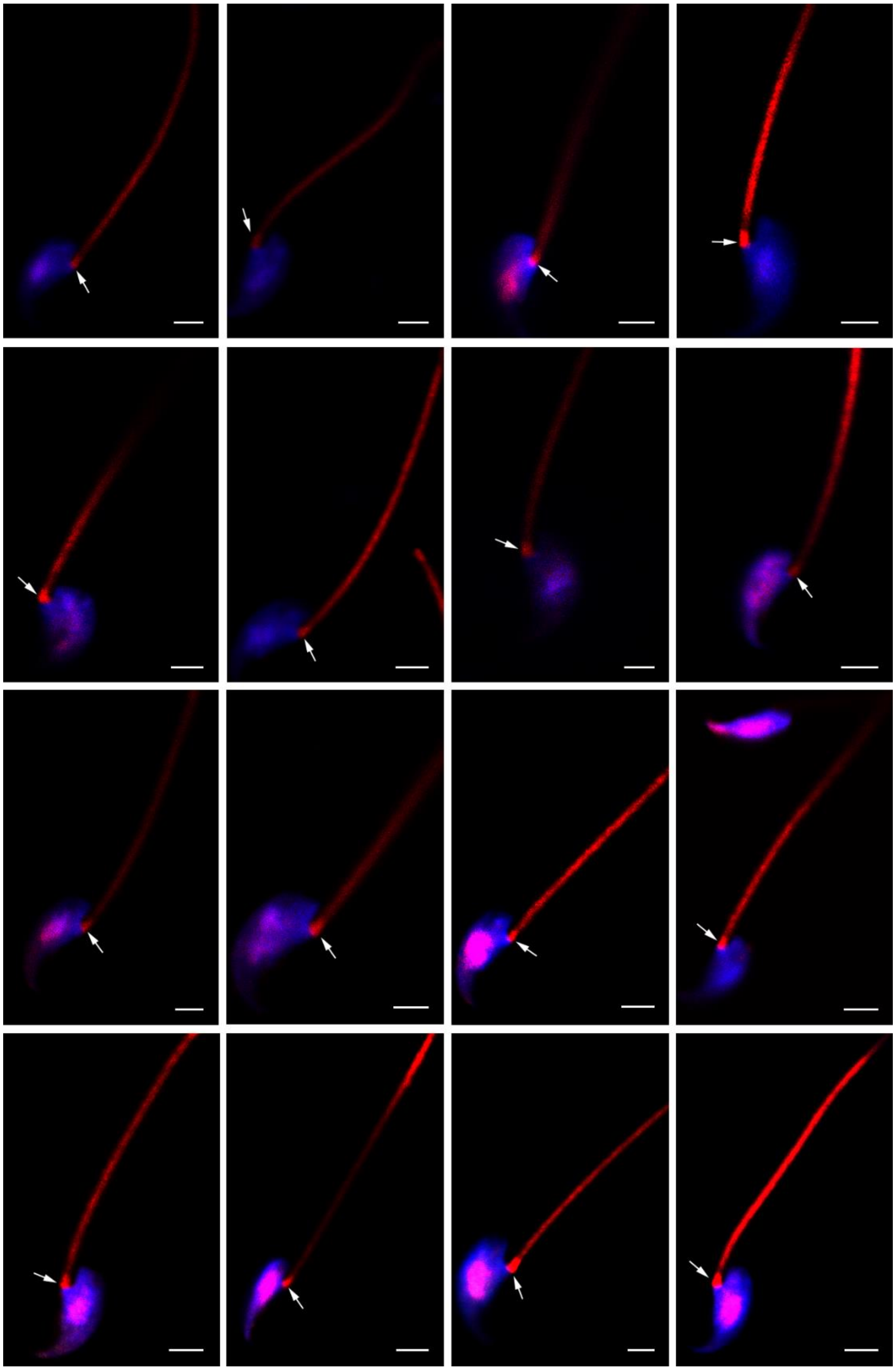

**Fig. S2-** Snapshot of 12 sperm after co-incubation with labeled *miR-34c-5p.* All show the red label localized at a high intensity at/near the centrosome (arrowed) after the miRNA internalization. Scale bars = 2 μm

**Fig. S3.** Videos showing the internalization of the labeled miRNAs in 3D reconstructions

Video S3a: 3D volume rendering of a series of maximum intensity projections of a sperm co-incubated with OVS loaded with labeled *miR-143-3p* and *miR-34c-5p*

Video S3b: 3D volume rendering of a series of maximum intensity projections of a control sperm co-incubated with medium with free labeled miRNAs.

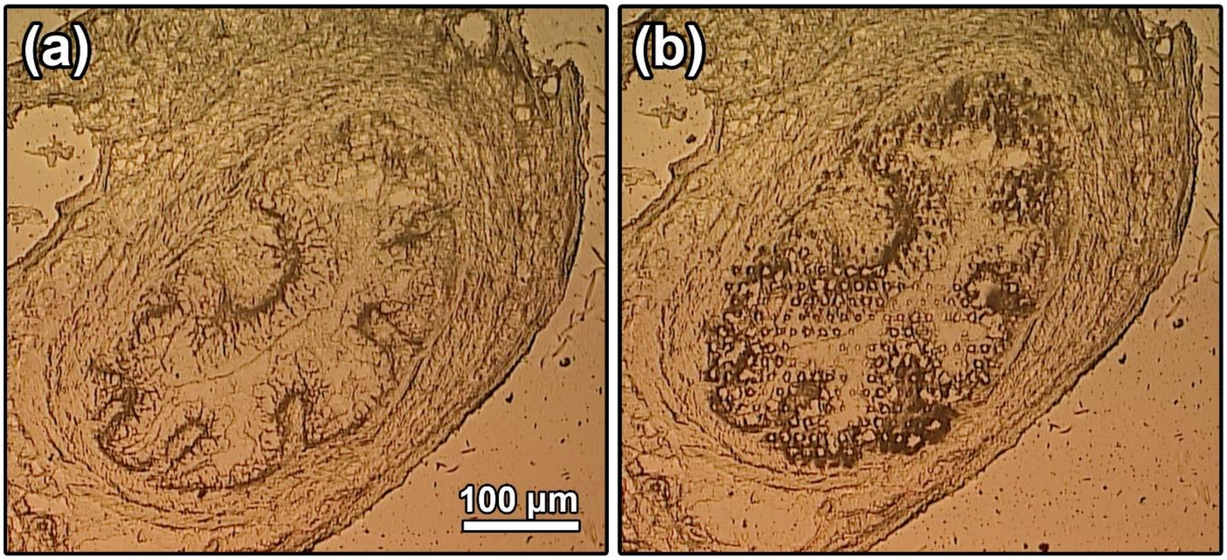


**Fig. S4- LM-Mediated isolation of epithelial cells from 20 µm thick sections of the oviductal tissue. a)** shows a cross-section of the oviductal tissue before and **b)** after microdissected.

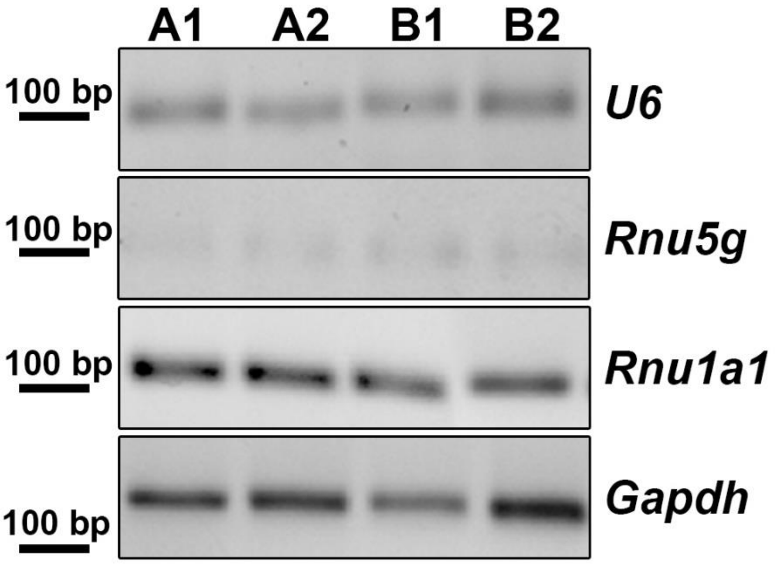


**Fig. S5- Selecting an endogenous control from four RNAs**. Four endogenous transcripts, *U6 snRNA*, *Rnu1a11A1*, *Run5g*, and *Gapdh*, were used to find the best internal control for the samples. There were two bioreplicates of purified OVS from pro/estrus (A) and met/diestrus (B). Gel electrophoresis of the RT-PCR products of ~ 100 bp are seen on 2% agarose gel stained with ethidium bromide. *U6 snRNA* was selected to normalize all samples, due to being consistent with all samples.

**Table S1**

| miRNA | A Bio1 Abundance (TP2M) | A Bio2 Abundance (TP2M) | B Bio1 Abundance (TP2M) | B Bio2 Abundance (TP2M) | Fold Change Bio1 (A/B) | Fold Change Bio2 (A/B) | Log2 Fold Change Bio1 | Log2 Fold Change Bio2 |
|---|---|---|---|---|---|---|---|---|
| **mmu-miR-451a** | 3.4416 | 271.2108 | 0.2952 | 11.8492 | 11.6585 | 22.8885 | 3.5433 | 4.5166 |
| **mmu-miR-1940** | 3.4416 | 2.0916 | 0.2952 | 0.2693 | 11.6585 | 7.7668 | 3.5433 | 2.9573 |
| **mmu-miR-488-3p** | 2.2944 | 2.4402 | 2.3616 | 0.5386 | 0.9715 | 4.5306 | -0.0416 | 2.1797 |
| **mmu-miR-3107-5p** | 425.6112 | 298.4016 | 319.9968 | 69.2101 | 1.3300 | 4.3115 | 0.4115 | 2.1082 |
| **mmu-miR-1843a-3p** | 8.7952 | 11.1552 | 5.0184 | 3.5009 | 1.7526 | 3.1864 | 0.8095 | 1.6719 |
| **mmu-miR-541-5p** | 57.36 | 32.7684 | 10.9224 | 11.3106 | 5.2516 | 2.8971 | 2.3928 | 1.5346 |
| **mmu-miR-350-3p** | 52.7712 | 68.6742 | 1.476 | 24.237 | 35.7528 | 2.8334 | 5.1600 | 1.5026 |
| **mmu-miR-30c-2-3p** | 4.5888 | 12.8982 | 4.428 | 4.5781 | 1.0363 | 2.8174 | 0.0515 | 1.4943 |
| **mmu-miR-15b-5p** | 8.4128 | 42.1806 | 1.476 | 17.7738 | 5.6997 | 2.3732 | 2.5109 | 1.2468 |
| **mmu-miR-185-5p** | 3.824 | 2.4402 | 1.1808 | 1.0772 | 3.2385 | 2.2653 | 1.6953 | 1.1797 |
| **mmu-miR-31-3p** | 45.888 | 36.603 | 16.8264 | 16.6966 | 2.7271 | 2.1922 | 1.4474 | 1.1324 |
| **mmu-miR-125a-3p** | 4.2064 | 3.486 | 0.5904 | 1.6158 | 7.1247 | 2.1574 | 2.8328 | 1.1093 |
| **mmu-miR-339-5p** | 2.2944 | 4.8804 | 0.5904 | 2.4237 | 3.8862 | 2.0136 | 1.9584 | 1.0098 |
| **mmu-miR-32-5p** | 2.6768 | 3.1374 | 0.8856 | 1.6158 | 3.0226 | 1.9417 | 1.5958 | 0.9573 |
| **mmu-miR-148b-5p** | 8.0304 | 7.3206 | 4.1328 | 3.7702 | 1.9431 | 1.9417 | 0.9584 | 0.9573 |
| **mmu-miR-34c-3p** | 11.472 | 13.944 | 2.3616 | 7.2711 | 4.8577 | 1.9177 | 2.2803 | 0.9394 |
| **mmu-miR-141-3p** | 71.8912 | 13.2468 | 76.4568 | 7.2711 | 0.9403 | 1.8218 | -0.0888 | 0.8654 |
| **mmu-miR-125a-5p** | 48.9472 | 133.8624 | 31.2912 | 73.7882 | 1.5642 | 1.8141 | 0.6455 | 0.8593 |
| **mmu-miR-301b-3p** | 5.3536 | 5.5776 | 2.6568 | 3.2316 | 2.0151 | 1.7260 | 1.0108 | 0.7874 |
| **mmu-miR-92a-3p** | 135.3696 | 162.099 | 94.464 | 93.9857 | 1.4330 | 1.7247 | 0.5191 | 0.7864 |
| **mmu-miR-92b-3p** | 18.7376 | 18.1272 | 9.7416 | 10.772 | 1.9235 | 1.6828 | 0.9437 | 0.7509 |
| **mmu-miR-30b-5p** | 728.8544 | 1358.4942 | 229.3704 | 826.751 | 3.1776 | 1.6432 | 1.6680 | 0.7165 |
| **mmu-miR-872-5p** | 47.4176 | 38.346 | 49.0032 | 23.4291 | 0.9676 | 1.6367 | -0.0475 | 0.7108 |
| **mmu-miR-191-5p** | 5069.8592 | 5414.1066 | 3666.384 | 3367.8658 | 1.3828 | 1.6076 | 0.4676 | 0.6849 |
| **mmu-miR-425-5p** | 3.824 | 7.6692 | 1.7712 | 4.8474 | 2.1590 | 1.5821 | 1.1104 | 0.6619 |
| **mmu-miR-154-5p** | 6.1184 | 11.5038 | 5.0184 | 7.5404 | 1.2192 | 1.5256 | 0.2859 | 0.6094 |
| **mmu-miR-511-3p** | 5.736 | 12.201 | 0.8856 | 8.079 | 6.4770 | 1.5102 | 2.6953 | 0.5948 |

| | | | | | | | | |
|---|---|---|---|---|---|---|---|---|
| **mmu-miR-106b-5p** | 53.1536 | 150.9438 | 32.7672 | 101.5261 | 1.6222 | 1.4867 | 0.6979 | 0.5722 |
| **mmu-miR-574-3p** | 306.6848 | 204.9768 | 81.18 | 140.8439 | 3.7778 | 1.4553 | 1.9176 | 0.5414 |
| **mmu-miR-376b-3p** | 4.5888 | 13.2468 | 2.952 | 9.1562 | 1.5545 | 1.4468 | 0.6364 | 0.5328 |
| **mmu-miR-200b-5p** | 14.5312 | 19.5216 | 10.0368 | 13.7343 | 1.4478 | 1.4214 | 0.5339 | 0.5073 |
| **mmu-miR-200b-3p** | 185.0816 | 179.8776 | 64.6488 | 126.8403 | 2.8629 | 1.4181 | 1.5175 | 0.5040 |
| **mmu-miR-375-3p** | 119.3088 | 176.043 | 79.1136 | 127.3789 | 1.5081 | 1.3820 | 0.5927 | 0.4668 |
| **mmu-miR-3068-5p** | 3.824 | 5.5776 | 1.7712 | 4.0395 | 2.1590 | 1.3808 | 1.1104 | 0.4655 |
| **mmu-miR-31-5p** | 258.8848 | 307.8138 | 200.4408 | 224.3269 | 1.2916 | 1.3722 | 0.3691 | 0.4565 |
| **mmu-miR-450b-5p** | 6.5008 | 12.8982 | 1.1808 | 9.4255 | 5.5054 | 1.3684 | 2.4609 | 0.4525 |
| **mmu-miR-30c-5p** | 1528.4528 | 901.4796 | 777.2616 | 662.2087 | 1.9665 | 1.3613 | 0.9756 | 0.4450 |
| **mmu-miR-200a-3p** | 185.464 | 299.796 | 113.9472 | 222.9804 | 1.6276 | 1.3445 | 0.7028 | 0.4271 |
| **mmu-miR-376c-3p** | 2.2944 | 13.5954 | 1.1808 | 10.2334 | 1.9431 | 1.3285 | 0.9584 | 0.4098 |
| **mmu-miR-34b-3p** | 36.7104 | 31.0254 | 21.5496 | 23.4291 | 1.7035 | 1.3242 | 0.7685 | 0.4051 |
| **mmu-miR-192-5p** | 2779.2832 | 3423.9492 | 3860.6256 | 2586.8958 | 0.7199 | 1.3236 | -0.4741 | 0.4044 |
| **mmu-miR-3068-3p** | 2.6768 | 2.4402 | 0.8856 | 1.8851 | 3.0226 | 1.2945 | 1.5958 | 0.3724 |
| **mmu-miR-221-5p** | 8.4128 | 8.0178 | 3.8376 | 6.1939 | 2.1922 | 1.2945 | 1.1324 | 0.3724 |
| **mmu-miR-411-5p** | 126.5744 | 344.7654 | 120.7368 | 268.4921 | 1.0483 | 1.2841 | 0.0681 | 0.3607 |
| **mmu-miR-34c-5p** | 3885.5664 | 2426.9532 | 1324.8576 | 1893.4483 | 2.9328 | 1.2818 | 1.5523 | 0.3581 |
| **mmu-miR-429-3p** | 331.9232 | 393.2208 | 175.644 | 308.8871 | 1.8897 | 1.2730 | 0.9182 | 0.3483 |
| **mmu-miR-351-5p** | 7.2656 | 19.8702 | 3.5424 | 15.6194 | 2.0510 | 1.2721 | 1.0364 | 0.3473 |
| **mmu-miR-582-3p** | 6.5008 | 14.2926 | 14.1696 | 11.5799 | 0.4588 | 1.2343 | -1.1241 | 0.3036 |
| **mmu-miR-16-5p** | 1014.5072 | 2352.7014 | 647.6688 | 1907.9905 | 1.5664 | 1.2331 | 0.6475 | 0.3023 |
| **mmu-miR-30e-3p** | 163.2848 | 240.8826 | 138.1536 | 195.5118 | 1.1819 | 1.2321 | 0.2411 | 0.3011 |
| **mmu-miR-125b-2-3p** | 5.736 | 12.8982 | 5.0184 | 10.5027 | 1.1430 | 1.2281 | 0.1928 | 0.2964 |
| **mmu-miR-19a-3p** | 31.3568 | 34.5114 | 14.76 | 28.2765 | 2.1244 | 1.2205 | 1.0871 | 0.2875 |
| **mmu-let-7g-5p** | 818.336 | 1559.6364 | 10722.2544 | 1280.2522 | 0.0763 | 1.2182 | -3.7118 | 0.2848 |
| **mmu-miR-204-5p** | 592.3376 | 151.2924 | 368.4096 | 124.4166 | 1.6078 | 1.2160 | 0.6851 | 0.2822 |
| **mmu-miR-138-5p** | 23.3264 | 57.8676 | 11.808 | 47.6661 | 1.9755 | 1.2140 | 0.9822 | 0.2798 |
| **mmu-miR-200c-3p** | 391.5776 | 381.0198 | 117.1944 | 314.0038 | 3.3413 | 1.2134 | 1.7404 | 0.2791 |
| **mmu-miR-301a-3p** | 279.5344 | 390.0834 | 143.172 | 322.6214 | 1.9524 | 1.2091 | 0.9653 | 0.2739 |
| **mmu-miR-421-3p** | 3.0592 | 4.5318 | 1.476 | 3.7702 | 2.0726 | 1.2020 | 1.0515 | 0.2654 |

| | | | | | | | | |
|---|---|---|---|---|---|---|---|---|
| **mmu-miR-182-5p** | 607.6336 | 270.8622 | 301.104 | 226.212 | 2.0180 | 1.1974 | 1.0129 | 0.2599 |
| **mmu-miR-30a-3p** | 136.8992 | 198.0048 | 104.5008 | 167.5046 | 1.3100 | 1.1821 | 0.3896 | 0.2413 |
| **mmu-miR-423-3p** | 96.3648 | 87.4986 | 39.5568 | 74.3268 | 2.4361 | 1.1772 | 1.2846 | 0.2354 |
| **mmu-miR-299a-5p** | 0.3824 | 3.486 | 5.6088 | 2.9623 | 0.0682 | 1.1768 | -3.8745 | 0.2349 |
| **mmu-miR-96-5p** | 39.0048 | 17.7786 | 11.2176 | 15.3501 | 3.4771 | 1.1582 | 1.7979 | 0.2119 |
| **mmu-miR-125b-5p** | 302.8608 | 717.4188 | 114.5376 | 622.8909 | 2.6442 | 1.1518 | 1.4028 | 0.2038 |
| **mmu-miR-425-3p** | 3.824 | 2.7888 | 3.5424 | 2.4237 | 1.0795 | 1.1506 | 0.1104 | 0.2024 |
| **mmu-miR-15a-5p** | 19.8848 | 34.86 | 2.6568 | 30.4309 | 7.4845 | 1.1455 | 2.9039 | 0.1960 |
| **mmu-miR-423-5p** | 24.0912 | 29.9796 | 7.6752 | 26.6607 | 3.1388 | 1.1245 | 1.6502 | 0.1693 |
| **mmu-miR-3102-3p** | 9.1776 | 11.5038 | 2.952 | 10.2334 | 3.1089 | 1.1241 | 1.6364 | 0.1688 |
| **mmu-miR-383-5p** | 5.736 | 4.5318 | 3.8376 | 4.0395 | 1.4947 | 1.1219 | 0.5798 | 0.1659 |
| **mmu-miR-10a-5p** | 4635.0704 | 6098.757 | 3266.388 | 5478.1006 | 1.4190 | 1.1133 | 0.5049 | 0.1548 |
| **mmu-miR-495-3p** | 6.5008 | 2.0916 | 2.6568 | 1.8851 | 2.4469 | 1.1095 | 1.2909 | 0.1500 |
| **mmu-miR-674-3p** | 4.5888 | 8.3664 | 2.3616 | 7.5404 | 1.9431 | 1.1095 | 0.9584 | 0.1500 |
| **mmu-miR-298-5p** | 4.2064 | 2.0916 | 6.4944 | 1.8851 | 0.6477 | 1.1095 | -0.6266 | 0.1500 |
| **mmu-miR-130b-3p** | 8.4128 | 8.0178 | 3.2472 | 7.2711 | 2.5908 | 1.1027 | 1.3734 | 0.1410 |
| **mmu-miR-1843b-5p** | 5.736 | 13.5954 | 4.7232 | 12.3878 | 1.2144 | 1.0975 | 0.2803 | 0.1342 |
| **mmu-miR-126a-3p** | 253.9136 | 736.5918 | 149.3712 | 674.8658 | 1.6999 | 1.0915 | 0.7654 | 0.1263 |
| **mmu-miR-150-5p** | 48.1824 | 112.9464 | 38.0808 | 103.6805 | 1.2653 | 1.0894 | 0.3394 | 0.1235 |
| **mmu-miR-26a-5p** | 19975.4288 | 35930.5506 | 6204.2184 | 33019.6809 | 3.2197 | 1.0882 | 1.6869 | 0.1219 |
| **mmu-miR-152-3p** | 444.3488 | 168.7224 | 93.5784 | 155.3861 | 4.7484 | 1.0858 | 2.2474 | 0.1188 |
| **mmu-miR-361-5p** | 44.3584 | 42.5292 | 13.8744 | 39.3178 | 3.1971 | 1.0817 | 1.6768 | 0.1133 |
| **mmu-miR-142-3p** | 27.5328 | 67.977 | 15.6456 | 63.2855 | 1.7598 | 1.0741 | 0.8154 | 0.1032 |
| **mmu-let-7a-1-3p** | 18.3552 | 24.7506 | 5.6088 | 23.1598 | 3.2726 | 1.0687 | 1.7104 | 0.0958 |
| **mmu-miR-1249-3p** | 11.0896 | 14.6412 | 2.952 | 13.7343 | 3.7566 | 1.0660 | 1.9094 | 0.0923 |
| **mmu-miR-135a-5p** | 42.064 | 42.1806 | 11.5128 | 39.5871 | 3.6537 | 1.0655 | 1.8693 | 0.0915 |
| **mmu-miR-326-3p** | 12.6192 | 30.3282 | 3.8376 | 28.5458 | 3.2883 | 1.0624 | 1.7173 | 0.0874 |
| **mmu-miR-148a-5p** | 62.7136 | 104.58 | 50.184 | 98.5638 | 1.2497 | 1.0610 | 0.3216 | 0.0855 |
| **mmu-miR-374b-5p** | 99.424 | 281.3202 | 47.5272 | 268.2228 | 2.0919 | 1.0488 | 1.0648 | 0.0688 |
| **mmu-miR-744-3p** | 3.0592 | 7.3206 | 1.476 | 7.0018 | 2.0726 | 1.0455 | 1.0515 | 0.0642 |
| **mmu-miR-1247-5p** | 8.4128 | 9.7608 | 1.1808 | 9.4255 | 7.1247 | 1.0356 | 2.8328 | 0.0504 |

| | | | | | | | | |
|---|---|---|---|---|---|---|---|---|
| **mmu-miR-30d-5p** | 159.0784 | 247.1574 | 97.1208 | 239.1384 | 1.6379 | 1.0335 | 0.7119 | 0.0476 |
| **mmu-miR-186-5p** | 385.0768 | 328.0326 | 141.696 | 318.3126 | 2.7176 | 1.0305 | 1.4423 | 0.0434 |
| **mmu-miR-652-3p** | 43.2112 | 44.6208 | 28.6344 | 43.3573 | 1.5091 | 1.0291 | 0.5937 | 0.0414 |
| **mmu-miR-205-5p** | 19.8848 | 34.86 | 112.4712 | 33.9318 | 0.1768 | 1.0274 | -2.4998 | 0.0389 |
| **mmu-let-7f-2-3p** | 3.0592 | 6.6234 | 1.476 | 6.4632 | 2.0726 | 1.0248 | 1.0515 | 0.0353 |
| **mmu-miR-18a-5p** | 37.4752 | 21.9618 | 2.952 | 21.544 | 12.6949 | 1.0194 | 3.6662 | 0.0277 |
| **mmu-miR-30d-3p** | 10.3248 | 24.7506 | 13.8744 | 24.5063 | 0.7442 | 1.0100 | -0.4263 | 0.0143 |
| **mmu-let-7i-3p** | 10.3248 | 28.5852 | 5.904 | 28.8151 | 1.7488 | 0.9920 | 0.8063 | -0.0116 |
| **mmu-miR-17-5p** | 60.4192 | 93.7734 | 48.4128 | 95.6015 | 1.2480 | 0.9809 | 0.3196 | -0.0279 |
| **mmu-miR-141-5p** | 16.8256 | 9.7608 | 6.1992 | 9.9641 | 2.7142 | 0.9796 | 1.4405 | -0.0297 |
| **mmu-miR-329-3p** | 3.0592 | 6.972 | 1.7712 | 7.2711 | 1.7272 | 0.9589 | 0.7884 | -0.0606 |
| **mmu-miR-194-5p** | 97.8944 | 130.0278 | 29.8152 | 136.2658 | 3.2834 | 0.9542 | 1.7152 | -0.0676 |
| **mmu-miR-455-3p** | 8.4128 | 9.7608 | 0.5904 | 10.2334 | 14.2493 | 0.9538 | 3.8328 | -0.0682 |
| **mmu-miR-100-5p** | 260.7968 | 220.6638 | 144.3528 | 232.4059 | 1.8067 | 0.9495 | 0.8533 | -0.0748 |
| **mmu-miR-139-5p** | 7.648 | 7.6692 | 1.476 | 8.079 | 5.1816 | 0.9493 | 2.3734 | -0.0751 |
| **mmu-miR-181a-5p** | 1659.2336 | 2402.2026 | 2419.4592 | 2553.7719 | 0.6858 | 0.9406 | -0.5442 | -0.0883 |
| **mmu-miR-148b-3p** | 469.5872 | 182.3178 | 73.2096 | 195.5118 | 6.4143 | 0.9325 | 2.6813 | -0.1008 |
| **mmu-miR-23b-3p** | 184.6992 | 180.9234 | 85.608 | 195.2425 | 2.1575 | 0.9267 | 1.1094 | -0.1099 |
| **mmu-miR-142-5p** | 511.6512 | 1236.4842 | 378.7416 | 1342.9991 | 1.3509 | 0.9207 | 0.4339 | -0.1192 |
| **mmu-miR-20a-5p** | 69.5968 | 140.4858 | 66.7152 | 152.9624 | 1.0432 | 0.9184 | 0.0610 | -0.1228 |
| **mmu-let-7d-3p** | 94.4528 | 109.4604 | 59.04 | 120.3771 | 1.5998 | 0.9093 | 0.6779 | -0.1372 |
| **mmu-miR-136-5p** | 2.2944 | 4.8804 | 2.3616 | 5.386 | 0.9715 | 0.9061 | -0.0416 | -0.1422 |
| **mmu-miR-99a-3p** | 7.648 | 8.0178 | 2.952 | 8.8869 | 2.5908 | 0.9022 | 1.3734 | -0.1485 |
| **mmu-miR-615-3p** | 6.1184 | 5.5776 | 2.0664 | 6.1939 | 2.9609 | 0.9005 | 1.5660 | -0.1512 |
| **mmu-miR-23a-3p** | 161.7552 | 199.3992 | 85.0176 | 221.6339 | 1.9026 | 0.8997 | 0.9280 | -0.1525 |
| **mmu-miR-223-3p** | 88.7168 | 147.1092 | 36.3096 | 164.0037 | 2.4433 | 0.8970 | 1.2889 | -0.1568 |
| **mmu-miR-151-3p** | 499.032 | 973.9884 | 597.78 | 1092.2808 | 0.8348 | 0.8917 | -0.2605 | -0.1654 |
| **mmu-miR-382-5p** | 9.9424 | 7.6692 | 3.8376 | 8.6176 | 2.5908 | 0.8899 | 1.3734 | -0.1682 |
| **mmu-miR-344-3p** | 13.7664 | 18.8244 | 7.38 | 21.2747 | 1.8654 | 0.8848 | 0.8995 | -0.1765 |
| **mmu-miR-434-5p** | 4.2064 | 5.229 | 1.476 | 5.9246 | 2.8499 | 0.8826 | 1.5109 | -0.1802 |
| **mmu-miR-195a-5p** | 523.888 | 980.6118 | 91.512 | 1111.4011 | 5.7248 | 0.8823 | 2.5172 | -0.1806 |

| | | | | | | | | |
|---|---|---|---|---|---|---|---|---|
| **mmu-miR-378a-3p** | 358.3088 | 568.5666 | 622.2816 | 646.8586 | 0.5758 | 0.8790 | -0.7964 | -0.1861 |
| **mmu-let-7a-5p** | 2054.6352 | 4470.0978 | 11931.984 | 5087.077 | 0.1722 | 0.8787 | -2.5379 | -0.1865 |
| **mmu-miR-296-5p** | 7.2656 | 8.0178 | 2.0664 | 9.1562 | 3.5161 | 0.8757 | 1.8140 | -0.1915 |
| **mmu-miR-181a-1-3p** | 43.2112 | 52.29 | 51.9552 | 59.7846 | 0.8317 | 0.8746 | -0.2659 | -0.1932 |
| **mmu-miR-676-3p** | 205.3488 | 305.7222 | 181.548 | 349.8207 | 1.1311 | 0.8739 | 0.1777 | -0.1944 |
| **mmu-miR-148a-3p** | 5049.2096 | 11374.818 | 4342.392 | 13037.8902 | 1.1628 | 0.8724 | 0.2176 | -0.1969 |
| **mmu-miR-24-2-5p** | 85.6576 | 131.4222 | 49.5936 | 152.9624 | 1.7272 | 0.8592 | 0.7884 | -0.2190 |
| **mmu-miR-25-3p** | 1765.9232 | 1919.7402 | 1305.3744 | 2238.9602 | 1.3528 | 0.8574 | 0.4360 | -0.2219 |
| **mmu-miR-155-5p** | 14.1488 | 24.402 | 6.1992 | 28.5458 | 2.2824 | 0.8548 | 1.1905 | -0.2263 |
| **mmu-miR-210-3p** | 186.2288 | 224.4984 | 92.1024 | 263.6447 | 2.0220 | 0.8515 | 1.0158 | -0.2319 |
| **mmu-let-7e-5p** | 403.8144 | 769.3602 | 790.8408 | 907.541 | 0.5106 | 0.8477 | -0.9697 | -0.2383 |
| **mmu-miR-669c-5p** | 5.3536 | 5.229 | 5.3136 | 6.1939 | 1.0075 | 0.8442 | 0.0108 | -0.2443 |
| **mmu-miR-212-5p** | 1.912 | 4.5318 | 4.1328 | 5.386 | 0.4626 | 0.8414 | -1.1120 | -0.2491 |
| **mmu-let-7d-5p** | 555.6272 | 900.4338 | 1185.5232 | 1071.0061 | 0.4687 | 0.8407 | -1.0933 | -0.2503 |
| **mmu-miR-484** | 26.0032 | 50.8956 | 19.188 | 60.8618 | 1.3552 | 0.8362 | 0.4385 | -0.2580 |
| **mmu-miR-434-3p** | 48.1824 | 141.5316 | 22.14 | 169.659 | 2.1763 | 0.8342 | 1.1219 | -0.2615 |
| **mmu-miR-214-3p** | 69.5968 | 82.9668 | 22.14 | 99.9103 | 3.1435 | 0.8304 | 1.6524 | -0.2681 |
| **mmu-miR-28a-5p** | 82.5984 | 60.6564 | 42.2136 | 73.5189 | 1.9567 | 0.8250 | 0.9684 | -0.2775 |
| **mmu-miR-337-3p** | 4.5888 | 2.4402 | 2.952 | 2.9623 | 1.5545 | 0.8238 | 0.6364 | -0.2797 |
| **mmu-let-7b-5p** | 906.288 | 1761.4758 | 850.4712 | 2149.2833 | 1.0656 | 0.8196 | 0.0917 | -0.2871 |
| **mmu-miR-127-3p** | 997.2992 | 754.0218 | 1194.6744 | 920.1981 | 0.8348 | 0.8194 | -0.2605 | -0.2873 |
| **mmu-miR-222-3p** | 73.8032 | 81.5724 | 60.2208 | 99.9103 | 1.2255 | 0.8165 | 0.2934 | -0.2926 |
| **mmu-miR-151-5p** | 204.2016 | 434.3556 | 120.1464 | 533.4833 | 1.6996 | 0.8142 | 0.7652 | -0.2966 |
| **mmu-miR-331-3p** | 3.4416 | 6.2748 | 1.1808 | 7.8097 | 2.9146 | 0.8035 | 1.5433 | -0.3157 |
| **mmu-miR-320-3p** | 92.9232 | 105.9744 | 92.6928 | 133.0342 | 1.0025 | 0.7966 | 0.0036 | -0.3281 |
| **mmu-miR-200a-5p** | 45.888 | 31.7226 | 27.7488 | 39.8564 | 1.6537 | 0.7959 | 0.7257 | -0.3293 |
| **mmu-miR-101b-3p** | 47.0352 | 118.1754 | 53.7264 | 148.9229 | 0.8755 | 0.7935 | -0.1919 | -0.3336 |
| **mmu-miR-99b-3p** | 8.4128 | 7.6692 | 6.4944 | 9.6948 | 1.2954 | 0.7911 | 0.3734 | -0.3381 |
| **mmu-miR-152-5p** | 40.5344 | 48.1068 | 26.8632 | 60.8618 | 1.5089 | 0.7904 | 0.5935 | -0.3393 |
| **mmu-miR-29b-3p** | 125.4272 | 254.8266 | 65.8296 | 323.16 | 1.9053 | 0.7885 | 0.9300 | -0.3427 |
| **mmu-miR-183-5p** | 724.648 | 38.346 | 673.9416 | 48.7433 | 1.0752 | 0.7867 | 0.1047 | -0.3461 |

| | | | | | | | | |
|---|---|---|---|---|---|---|---|---|
| **mmu-miR-93-5p** | 322.7456 | 553.5768 | 327.0816 | 705.8353 | 0.9867 | 0.7843 | -0.0193 | -0.3505 |
| **mmu-miR-744-5p** | 23.7088 | 23.3562 | 14.4648 | 29.8923 | 1.6391 | 0.7813 | 0.7129 | -0.3560 |
| **mmu-miR-133a-3p** | 147.9888 | 142.926 | 72.6192 | 183.3933 | 2.0379 | 0.7793 | 1.0271 | -0.3597 |
| **mmu-miR-381-3p** | 60.8016 | 126.1932 | 89.4456 | 162.9265 | 0.6798 | 0.7745 | -0.5569 | -0.3686 |
| **mmu-miR-146b-5p** | 159.4608 | 56.8218 | 166.788 | 73.5189 | 0.9561 | 0.7729 | -0.0648 | -0.3717 |
| **mmu-miR-218-5p** | 45.1232 | 90.2874 | 70.5528 | 116.8762 | 0.6396 | 0.7725 | -0.6448 | -0.3724 |
| **mmu-miR-872-3p** | 113.1904 | 232.1676 | 34.8336 | 300.8081 | 3.2495 | 0.7718 | 1.7002 | -0.3737 |
| **mmu-miR-140-5p** | 21.7968 | 49.1526 | 15.3504 | 64.0934 | 1.4199 | 0.7669 | 0.5058 | -0.3829 |
| **mmu-miR-328-3p** | 75.7152 | 58.2162 | 63.7632 | 75.9426 | 1.1874 | 0.7666 | 0.2479 | -0.3835 |
| **mmu-miR-1843a-5p** | 29.0624 | 65.8854 | 34.5384 | 86.4453 | 0.8415 | 0.7622 | -0.2490 | -0.3918 |
| **mmu-miR-503-3p** | 6.1184 | 6.972 | 0.8856 | 9.1562 | 6.9088 | 0.7615 | 2.7884 | -0.3932 |
| **mmu-miR-532-5p** | 147.224 | 129.3306 | 183.6144 | 169.9283 | 0.8018 | 0.7611 | -0.3187 | -0.3939 |
| **mmu-miR-365-3p** | 46.2704 | 128.6334 | 50.184 | 169.1204 | 0.9220 | 0.7606 | -0.1171 | -0.3948 |
| **mmu-let-7e-3p** | 5.736 | 5.9262 | 4.1328 | 7.8097 | 1.3879 | 0.7588 | 0.4729 | -0.3982 |
| **mmu-miR-126a-5p** | 3952.4864 | 6138.846 | 3109.932 | 8093.0036 | 1.2709 | 0.7585 | 0.3459 | -0.3987 |
| **mmu-miR-29a-3p** | 560.9808 | 912.2862 | 176.8248 | 1203.5017 | 3.1725 | 0.7580 | 1.6656 | -0.3997 |
| **mmu-miR-467d-3p** | 1.1472 | 2.4402 | 2.3616 | 3.2316 | 0.4858 | 0.7551 | -1.0416 | -0.4052 |
| **mmu-miR-149-5p** | 77.6272 | 36.9516 | 97.7112 | 49.0126 | 0.7945 | 0.7539 | -0.3320 | -0.4075 |
| **mmu-miR-676-5p** | 29.8272 | 22.659 | 28.044 | 30.1616 | 1.0636 | 0.7513 | 0.0889 | -0.4126 |
| **mmu-miR-672-5p** | 90.6288 | 179.8776 | 113.3568 | 239.9463 | 0.7995 | 0.7497 | -0.3228 | -0.4157 |
| **mmu-miR-21a-5p** | 25239.5472 | 21749.154 | 20055.888 | 29025.154 | 1.2585 | 0.7493 | 0.3317 | -0.4163 |
| **mmu-miR-10b-5p** | 10203.1968 | 14401.7118 | 8150.472 | 19249.2947 | 1.2519 | 0.7482 | 0.3241 | -0.4186 |
| **mmu-miR-127-5p** | 4.9712 | 5.5776 | 0.5904 | 7.5404 | 8.4201 | 0.7397 | 3.0738 | -0.4350 |
| **mmu-miR-450a-5p** | 18.7376 | 12.5496 | 4.428 | 16.9659 | 4.2316 | 0.7397 | 2.0812 | -0.4350 |
| **mmu-miR-190a-3p** | 4.5888 | 5.9262 | 8.5608 | 8.079 | 0.5360 | 0.7335 | -0.8996 | -0.4471 |
| **mmu-miR-101a-3p** | 396.1664 | 1127.0238 | 436.6008 | 1545.782 | 0.9074 | 0.7291 | -0.1402 | -0.4558 |
| **mmu-miR-455-5p** | 17.208 | 31.7226 | 21.2544 | 43.8959 | 0.8096 | 0.7227 | -0.3047 | -0.4686 |
| **mmu-miR-501-3p** | 26.3856 | 1.743 | 23.3208 | 2.4237 | 1.1314 | 0.7191 | 0.1781 | -0.4756 |
| **mmu-miR-379-5p** | 28.68 | 11.1552 | 18.8928 | 15.6194 | 1.5180 | 0.7142 | 0.6022 | -0.4856 |
| **mmu-miR-34b-5p** | 85.6576 | 85.0584 | 63.1728 | 119.2999 | 1.3559 | 0.7130 | 0.4393 | -0.4881 |
| **mmu-miR-299a-3p** | 2.2944 | 3.8346 | 1.7712 | 5.386 | 1.2954 | 0.7120 | 0.3734 | -0.4901 |

| | | | | | | | | |
|---|---|---|---|---|---|---|---|---|
| **mmu-let-7c-5p** | 6786.4528 | 11957.3286 | 14089.896 | 16862.2195 | 0.4817 | 0.7091 | -1.0539 | -0.4959 |
| **mmu-miR-342-3p** | 398.4608 | 85.407 | 128.7072 | 121.4543 | 3.0959 | 0.7032 | 1.6303 | -0.5080 |
| **mmu-miR-322-5p** | 148.3712 | 294.9156 | 61.6968 | 422.5317 | 2.4048 | 0.6980 | 1.2659 | -0.5188 |
| **mmu-miR-29c-3p** | 28.68 | 65.5368 | 30.7008 | 93.9857 | 0.9342 | 0.6973 | -0.0982 | -0.5201 |
| **mmu-miR-497-5p** | 68.4496 | 172.2084 | 15.0552 | 249.6411 | 4.5466 | 0.6898 | 2.1848 | -0.5357 |
| **mmu-miR-1839-5p** | 104.7776 | 106.6716 | 213.7248 | 154.8475 | 0.4902 | 0.6889 | -1.0284 | -0.5377 |
| **mmu-miR-19b-3p** | 112.808 | 128.2848 | 67.0104 | 186.8942 | 1.6834 | 0.6864 | 0.7514 | -0.5429 |
| **mmu-miR-184-3p** | 33.2688 | 15.3384 | 27.4536 | 22.3519 | 1.2118 | 0.6862 | 0.2772 | -0.5432 |
| **mmu-miR-103-3p** | 684.8784 | 656.4138 | 773.1288 | 961.9396 | 0.8859 | 0.6824 | -0.1749 | -0.5513 |
| **mmu-miR-195a-3p** | 14.5312 | 23.7048 | 15.9408 | 34.7397 | 0.9116 | 0.6824 | -0.1336 | -0.5514 |
| **mmu-miR-99a-5p** | 460.0272 | 356.2692 | 761.0256 | 524.3271 | 0.6045 | 0.6795 | -0.7262 | -0.5575 |
| **mmu-miR-190a-5p** | 4.9712 | 9.0636 | 2.0664 | 13.465 | 2.4057 | 0.6731 | 1.2665 | -0.5711 |
| **mmu-miR-582-5p** | 3.0592 | 18.8244 | 3.2472 | 28.0072 | 0.9421 | 0.6721 | -0.0860 | -0.5732 |
| **mmu-miR-27b-5p** | 13.7664 | 25.4478 | 21.5496 | 37.9713 | 0.6388 | 0.6702 | -0.6465 | -0.5774 |
| **mmu-miR-22-3p** | 5982.648 | 7482.0018 | 10954.5768 | 11216.0757 | 0.5461 | 0.6671 | -0.8727 | -0.5841 |
| **mmu-miR-335-5p** | 6.1184 | 12.5496 | 2.0664 | 18.851 | 2.9609 | 0.6657 | 1.5660 | -0.5870 |
| **mmu-miR-199a-3p** | 1659.9984 | 2924.0568 | 1147.1472 | 4397.3997 | 1.4471 | 0.6650 | 0.5331 | -0.5887 |
| **mmu-let-7f-5p** | 11215.0272 | 18096.8718 | 32925.7224 | 27305.6735 | 0.3406 | 0.6628 | -1.5538 | -0.5935 |
| **mmu-miR-500-3p** | 13.0016 | 8.0178 | 10.6272 | 12.1185 | 1.2234 | 0.6616 | 0.2909 | -0.5959 |
| **mmu-miR-362-3p** | 33.2688 | 36.603 | 12.3984 | 55.7451 | 2.6833 | 0.6566 | 1.4240 | -0.6069 |
| **mmu-miR-130a-3p** | 107.8368 | 159.6588 | 70.5528 | 243.9858 | 1.5285 | 0.6544 | 0.6121 | -0.6118 |
| **mmu-miR-503-5p** | 2.6768 | 5.5776 | 0.5904 | 8.6176 | 4.5339 | 0.6472 | 2.1807 | -0.6276 |
| **mmu-miR-140-3p** | 2.2944 | 8.3664 | 3.8376 | 12.9264 | 0.5979 | 0.6472 | -0.7421 | -0.6276 |
| **mmu-miR-1983** | 3.0592 | 2.4402 | 6.7896 | 3.7702 | 0.4506 | 0.6472 | -1.1502 | -0.6276 |
| **mmu-miR-181c-3p** | 64.2432 | 66.5826 | 100.0728 | 103.4112 | 0.6420 | 0.6439 | -0.6394 | -0.6352 |
| **mmu-miR-1198-5p** | 25.6208 | 13.5954 | 7.38 | 21.544 | 3.4717 | 0.6311 | 1.7956 | -0.6642 |
| **mmu-miR-199b-5p** | 294.0656 | 880.5636 | 84.132 | 1395.5126 | 3.4953 | 0.6310 | 1.8054 | -0.6643 |
| **mmu-miR-146a-5p** | 1086.7808 | 417.2742 | 648.5544 | 663.5552 | 1.6757 | 0.6288 | 0.7448 | -0.6692 |
| **mmu-miR-22-5p** | 3.4416 | 23.7048 | 2.6568 | 37.9713 | 1.2954 | 0.6243 | 0.3734 | -0.6797 |
| **mmu-miR-26b-5p** | 949.1168 | 797.5968 | 1326.3336 | 1279.7136 | 0.7156 | 0.6233 | -0.4828 | -0.6821 |
| **mmu-miR-27b-3p** | 12894.528 | 14036.0304 | 16209.1368 | 22528.8301 | 0.7955 | 0.6230 | -0.3300 | -0.6826 |

| | | | | | | | | |
|---|---|---|---|---|---|---|---|---|
| **mmu-let-7i-5p** | 4287.8512 | 7342.2132 | 6255.288 | 11801.2646 | 0.6855 | 0.6222 | -0.5448 | -0.6847 |
| **mmu-miR-28a-3p** | 236.7056 | 244.3686 | 275.4216 | 393.7166 | 0.8594 | 0.6207 | -0.2185 | -0.6881 |
| **mmu-miR-340-5p** | 2152.1472 | 2762.3064 | 4417.668 | 4484.9222 | 0.4872 | 0.6159 | -1.0375 | -0.6992 |
| **mmu-miR-128-3p** | 17.9728 | 13.2468 | 13.8744 | 21.544 | 1.2954 | 0.6149 | 0.3734 | -0.7016 |
| **mmu-miR-130a-5p** | 5.3536 | 3.1374 | 0.2952 | 5.1167 | 18.1355 | 0.6132 | 4.1807 | -0.7056 |
| **mmu-miR-9-5p** | 2.2944 | 3.1374 | 7.38 | 5.1167 | 0.3109 | 0.6132 | -1.6855 | -0.7056 |
| **mmu-miR-410-3p** | 68.4496 | 91.3332 | 87.3792 | 150.808 | 0.7834 | 0.6056 | -0.3522 | -0.7235 |
| **mmu-miR-30a-5p** | 494.0608 | 708.3552 | 474.0912 | 1170.6471 | 1.0421 | 0.6051 | 0.0595 | -0.7248 |
| **mmu-miR-99b-5p** | 2536.4592 | 429.8238 | 1636.2936 | 712.5678 | 1.5501 | 0.6032 | 0.6324 | -0.7293 |
| **mmu-miR-324-5p** | 6.5008 | 14.2926 | 6.4944 | 23.6984 | 1.0010 | 0.6031 | 0.0014 | -0.7295 |
| **mmu-miR-378a-5p** | 20.6496 | 29.9796 | 9.1512 | 49.8205 | 2.2565 | 0.6018 | 1.1741 | -0.7328 |
| **mmu-miR-181d-5p** | 275.328 | 234.9564 | 243.2448 | 390.485 | 1.1319 | 0.6017 | 0.1787 | -0.7329 |
| **mmu-miR-181b-5p** | 171.6976 | 247.8546 | 133.4304 | 414.9913 | 1.2868 | 0.5973 | 0.3638 | -0.7436 |
| **mmu-miR-221-3p** | 262.3264 | 257.964 | 188.928 | 432.2265 | 1.3885 | 0.5968 | 0.4735 | -0.7446 |
| **mmu-miR-1247-3p** | 4.2064 | 5.9262 | 5.904 | 9.9641 | 0.7125 | 0.5948 | -0.4891 | -0.7496 |
| **mmu-miR-30e-5p** | 68.4496 | 107.0202 | 62.5824 | 181.7775 | 1.0938 | 0.5887 | 0.1293 | -0.7643 |
| **mmu-miR-29a-5p** | 4.5888 | 3.486 | 1.476 | 5.9246 | 3.1089 | 0.5884 | 1.6364 | -0.7651 |
| **mmu-miR-132-3p** | 12.6192 | 13.944 | 9.7416 | 23.9677 | 1.2954 | 0.5818 | 0.3734 | -0.7814 |
| **mmu-miR-199a-5p** | 341.8656 | 573.7956 | 182.4336 | 991.024 | 1.8739 | 0.5790 | 0.9061 | -0.7884 |
| **mmu-miR-181c-5p** | 1173.5856 | 2036.5212 | 2130.4584 | 3525.6756 | 0.5509 | 0.5776 | -0.8602 | -0.7918 |
| **mmu-miR-341-3p** | 10.3248 | 11.5038 | 13.8744 | 19.9282 | 0.7442 | 0.5773 | -0.4263 | -0.7927 |
| **mmu-miR-669a-5p** | 19.8848 | 3.8346 | 13.8744 | 6.7325 | 1.4332 | 0.5696 | 0.5192 | -0.8121 |
| **mmu-miR-125b-1-3p** | 4.2064 | 5.9262 | 2.952 | 10.5027 | 1.4249 | 0.5643 | 0.5109 | -0.8256 |
| **mmu-miR-98-5p** | 1186.9696 | 2292.045 | 516.8952 | 4070.4695 | 2.2963 | 0.5631 | 1.1993 | -0.8286 |
| **mmu-miR-24-3p** | 47.0352 | 71.1144 | 22.4352 | 127.1096 | 2.0965 | 0.5595 | 1.0680 | -0.8379 |
| **mmu-miR-27a-3p** | 2416.3856 | 884.7468 | 3305.0592 | 1623.6097 | 0.7311 | 0.5449 | -0.4518 | -0.8759 |
| **mmu-miR-449a-5p** | 47.0352 | 14.6412 | 48.1176 | 26.93 | 0.9775 | 0.5437 | -0.0328 | -0.8792 |
| **mmu-miR-196b-3p** | 3.0592 | 3.1374 | 0.2952 | 5.9246 | 10.3631 | 0.5296 | 3.3734 | -0.9171 |
| **mmu-miR-708-3p** | 42.8288 | 50.1984 | 33.6528 | 95.0629 | 1.2727 | 0.5281 | 0.3479 | -0.9212 |
| **mmu-miR-202-5p** | 134.9872 | 95.5164 | 69.372 | 181.2389 | 1.9458 | 0.5270 | 0.9604 | -0.9241 |
| **mmu-miR-143-3p** | 111893.6816 | 102954.4782 | 141006.1176 | 195677.1502 | 0.7935 | 0.5261 | -0.3336 | -0.9265 |

| | | | | | | | | |
|---|---|---|---|---|---|---|---|---|
| **mmu-miR-196b-5p** | 3322.6736 | 1867.1016 | 3727.4904 | 3571.4566 | 0.8914 | 0.5228 | -0.1659 | -0.9357 |
| **mmu-miR-193b-3p** | 69.9792 | 78.7836 | 82.3608 | 151.6159 | 0.8497 | 0.5196 | -0.2350 | -0.9445 |
| **mmu-miR-26b-3p** | 5.3536 | 5.5776 | 3.5424 | 10.772 | 1.5113 | 0.5178 | 0.5958 | -0.9496 |
| **mmu-miR-203-3p** | 44.7408 | 71.8116 | 180.3672 | 138.6895 | 0.2481 | 0.5178 | -2.0113 | -0.9496 |
| **mmu-miR-107-3p** | 11.0896 | 8.0178 | 14.1696 | 15.6194 | 0.7826 | 0.5133 | -0.3536 | -0.9621 |
| **mmu-miR-34a-5p** | 54.3008 | 54.3816 | 57.2688 | 106.3735 | 0.9482 | 0.5112 | -0.0768 | -0.9679 |
| **mmu-miR-21a-3p** | 4.2064 | 1.743 | 4.7232 | 3.5009 | 0.8906 | 0.4979 | -0.1672 | -1.0062 |
| **mmu-miR-129-2-3p** | 9.1776 | 10.1094 | 2.3616 | 20.4668 | 3.8862 | 0.4939 | 1.9584 | -1.0176 |
| **mmu-miR-409-3p** | 19.5024 | 17.7786 | 18.5976 | 36.0862 | 1.0487 | 0.4927 | 0.0685 | -1.0213 |
| **mmu-miR-30b-3p** | 6.1184 | 2.0916 | 7.9704 | 4.3088 | 0.7676 | 0.4854 | -0.3815 | -1.0427 |
| **mmu-miR-193a-3p** | 172.08 | 247.1574 | 129.5928 | 523.7885 | 1.3279 | 0.4719 | 0.4091 | -1.0836 |
| **mmu-miR-300-3p** | 9.56 | 9.4122 | 7.6752 | 20.1975 | 1.2456 | 0.4660 | 0.3168 | -1.1016 |
| **mmu-miR-3057-5p** | 3.824 | 1.743 | 3.2472 | 3.7702 | 1.1776 | 0.4623 | 0.2359 | -1.1131 |
| **mmu-miR-135b-5p** | 3.4416 | 2.4402 | 5.904 | 5.386 | 0.5829 | 0.4531 | -0.7786 | -1.1422 |
| **mmu-miR-338-3p** | 0.7648 | 4.1832 | 5.0184 | 9.4255 | 0.1524 | 0.4438 | -2.7141 | -1.1720 |
| **mmu-miR-196a-5p** | 252.0016 | 152.6868 | 282.2112 | 346.0505 | 0.8930 | 0.4412 | -0.1633 | -1.1804 |
| **mmu-miR-708-5p** | 217.968 | 178.4832 | 178.596 | 409.0667 | 1.2205 | 0.4363 | 0.2874 | -1.1965 |
| **mmu-miR-214-5p** | 87.5696 | 53.6844 | 145.8288 | 126.8403 | 0.6005 | 0.4232 | -0.7358 | -1.2404 |
| **mmu-miR-322-3p** | 3.824 | 3.486 | 1.7712 | 8.3483 | 2.1590 | 0.4176 | 1.1104 | -1.2599 |
| **mmu-miR-106b-3p** | 6.1184 | 2.7888 | 11.2176 | 6.7325 | 0.5454 | 0.4142 | -0.8745 | -1.2715 |
| **mmu-miR-335-3p** | 8.0304 | 9.0636 | 6.4944 | 22.6212 | 1.2365 | 0.4007 | 0.3063 | -1.3195 |
| **mmu-miR-542-3p** | 11.0896 | 6.972 | 8.5608 | 18.851 | 1.2954 | 0.3698 | 0.3734 | -1.4350 |
| **mmu-miR-129-5p** | 9.9424 | 6.2748 | 8.2656 | 17.5045 | 1.2029 | 0.3585 | 0.2665 | -1.4801 |
| **mmu-miR-145a-5p** | 3002.9872 | 4793.5986 | 1614.4488 | 13542.2891 | 1.8601 | 0.3540 | 0.8954 | -1.4983 |
| **mmu-miR-700-5p** | 4.9712 | 1.3944 | 2.0664 | 4.0395 | 2.4057 | 0.3452 | 1.2665 | -1.5345 |
| **mmu-miR-134-5p** | 5.736 | 5.229 | 10.332 | 19.1203 | 0.5552 | 0.2735 | -0.8490 | -1.8705 |
| **mmu-miR-377-3p** | 0.3824 | 0.6972 | 2.3616 | 2.9623 | 0.1619 | 0.2354 | -2.6266 | -2.0871 |
| **mmu-miR-143-5p** | 34.416 | 22.3104 | 84.7224 | 95.6015 | 0.4062 | 0.2334 | -1.2997 | -2.0993 |

**Table S2.** Rates of cytoplasmic droplets (CD) after sperm co-incubation with HTF (as control) and OVS for 30 min and 3h. Using $\chi 2$ analysis, there is no significant ($P > 0.05$) difference between samples after co-incubation with OVS and HTF for different time periods.

| **Incubation time** | **Number (%)** | | |
|---|---|---|---|
| | **Status** | **HTF** | **OVS** |
| **30 min** | **No CD** | 162 | 194 |
| | **CD** | 11 (6.4) | 18 (8.5) |
| | **Total** | 173 | 212 |
| **3 h** | **No CD** | 199 | 184 |
| | **CD** | 10 (4.8) | 15 (7.5) |
| | **Total** | 209 | 199 |
| **Grand Total** | **No CD** | 361 | 378 |
| | **CD** | 21 (5.8) | 33 (8.7) |
| | **Total** | 382 | 411 |

## Table S3. Primers used for amplification of reverse-transcribed RNAs.

| Assay Name | U6 as an endogenous control and selected miRNA Sequence (5' to 3') | Accession Number (Exiqon Inc.) |
|---|---|---|
| *U6 snRNA (hsa, mmu)* | GUGCUCGCUUCGGCAGCACAUAUACUAAAAUUGGAA CGAUACAGAGAAGAUUAGCAUGGCCCCUGCGCAAGG AUGACACGCAAAUUCGUGAAGCGUUCCAUAUUUUU | 203907 |
| *miR-143-3p* | UGAGAUGAAGCACUGUAGCUC | 205992 |
| *miR-34c-5p* | AGGCAGUGUAGUUAGCUGAUUGC | 205659 |
| *miR-22-3p* | AAGCUGCCAGUUGAAGAACUGU | 204606 |
| *let-7a-5p* | UGAGGUAGUAGGUUGUAUAGUU | 205727 |

## Table S4. Primers for conventional RT-PCR.

| Gene | Forward Primer Sequence | Reverse Primer Sequence | Product Size (kbp) |
|---|---|---|---|
| *U6 snRNA* | CGCTTCGGCAGCACATATAC | AAATATGGAACGCTTCACGA | 100 |
| *Rnu5g* | CTCTGGTTTCTCTTCAGATCGT | TTGTCAAGACAAGGCCTCAA | 109 |
| *Rnu1a1* | GATCACGAAGGTGGTTTTCC | CAGTCCCCCACTACCACAAA | 119 |
| *Gapdh* | CCGCATCTTCTTGTGCAGT | GAATTTGCCCGTGAGTGGAGT | 204 |
